# A comprehensive dynamic immune acetylproteomics profiling induced by *Puccinia polysora* in maize

**DOI:** 10.1186/s12870-022-03964-4

**Published:** 2022-12-24

**Authors:** Jianfei Guo, Zhigang Ma, Ce Deng, Junqiang Ding, Yuxiao Chang

**Affiliations:** 1 Shenzhen Branch, Guangdong Laboratory of Lingnan Modern Agriculture, Genome Analysis Laboratory of the Ministry of Agriculture and Rural Affairs, Agricultural Genomics Institute at Shenzhen, Chinese Academy of Agricultural Sciences, Shenzhen, 518120 China; 2 State Key Laboratory of Crop Stress Adaptation and Improvement, School of Life Sciences, Henan University, Kaifeng, 475004 China; 3 Shenzhen Research Institute of Henan university, Shenzhen, 518000 China; 4 College of Agronomy, Henan Agricultural University, Zhengzhou, 450046 China; 5 The State Key Laboratory of Wheat and Maize Crop Science and Center for Crop Genome Engineering, Henan Agricultural University, Zhengzhou, 450046 China

**Keywords:** Southern corn rust (SCR), Mass spectrometry, Quantitative proteomics, Lysine acetylation, Plant-pathogen interaction, Immune response, Puccinia polysora

## Abstract

Lysine-ε-acetylation (Kac) is a reversible post-translational modification that plays important roles during plant-pathogen interactions. Some pathogens can deliver secreted effectors encoding acetyltransferases or deacetylases into host cell to directly modify acetylation of host proteins. However, the function of these acetylated host proteins in plant-pathogen defense remains to be determined. Employing high-resolution tandem mass spectrometry, we analyzed protein abundance and lysine acetylation changes in maize infected with *Puccinia polysora* (*P. polysora*) at 0 h, 12 h, 24 h, 48 h and 72 h. A total of 7412 Kac sites from 4697 proteins were identified, and 1732 Kac sites from 1006 proteins were quantified. Analyzed the features of lysine acetylation, we found that Kac is ubiquitous in cellular compartments and preferentially targets lysine residues in the -F/W/Y-X-X-K (ac)-N/S/T/P/Y/G- motif of the protein, this Kac motif contained proteins enriched in basic metabolism and defense-associated pathways during fungal infection. Further analysis of acetylproteomics data indicated that maize regulates cellular processes in response to *P. polysora* infection by altering Kac levels of histones and non-histones. In addition, acetylation of pathogen defense-related proteins presented converse patterns in signaling transduction, defense response, cell wall fortification, ROS scavenging, redox reaction and proteostasis. Our results provide informative resources for studying protein acetylation in plant-pathogen interactions, not only greatly extending the understanding on the roles of acetylation in vivo, but also providing a comprehensive dynamic pattern of Kac modifications in the process of plant immune response.

**Supplementary Information:**

The online version contains supplementary material available at 10.1186/s12870-022-03964-4.

## Introduction

Lysine-ε-acetylation (Kac) is a conserved post-translational modification (PTM) that was first discovered on histone proteins in 1964 [1], and occurs widely on non-histone in distinct organisms [2, 3]. A few studies showed that Kac of non-histone is involved in the regulation of a range of biological processes [4]. Kac is enzymatically regulated by lysine acetyltransferases (KATs), which catalyze the transfer of an acetyl group from coenzyme A (CoA) to specific lysine residues at the amino terminus of the protein, and by lysine deacetylases (KDACs), which remove the acetyl group from lysine [5]. Kac plays important roles in plant-pathogen interactions [6]. Some pathogens can secrete effector proteins encoding KATs or KDACs to directly modify the acetylation of host proteins to alter immunity, such as *Pseudomonas syringae* type III effector (T3E) HopZ1a suppresses cell wall-mediated defense by acetylating tubulin [7] and HopZ3 inhibits plant defense by acetylating multiple members of the RPM1 immune complex [8]. The deacetylase inhibitor HC-toxin (HCT) generated by the filamentous fungus *Cochliobolus carbonum* suppresses host defense through altering protein acetylation [9]. The polysaccharide deacetylase Pst_13661 from *Puccinia striiformis f.* sp. *tritici* suppresses chitin-induced plant defense by modifying the fungal cell wall [10]. The acetyltransferase PopP2 of *Ralstonia solanacearum* restrains immunity response by acetylating RRS1 and WRKY transcription factors to abolish their DNA binding activity [11–13]. Meanwhile, the acetyltransferase activity of PopP2 was antagonistically regulated by immune response proteins PAD4 and EDS1 [14].

Unlike animal, sessile plants lack a sophisticated adaptive immune system. To cope with potential microbial invasions, plants have evolved elaborate systemic physical barriers and innate immune systems to provide immunity against pathogens attack [15, 16]. The waxy cuticle and cell wall provide physical and mechanical barriers for plant cells to protect against pathogens [17]. Innate immune system is an essential part of the defense of plants against microbial attack [18]. Plant uses a suite of cell surface localized pattern recognition receptors to detect various pathogen-associated molecular patterns (PAMPs) triggered immunity (PTI), and employs intracellular resistance (R) proteins to recognize pathogen secreted effectors and thus induce effector-triggered immunity (ETI) [19]. Activation of the receptors by PTI and ETI can trigger a number of key signaling modules, including rapid phosphorylation of receptor-like cytoplasmic kinases (RLCKs), plasma membrane (PM) depolarization caused by an influx of calcium and H^+^ across PM, activation of calcium-dependent kinases (CDPKs), mitogen-activated protein kinase (MAPK) cascades, a burst of reaction oxygen species (ROS), stomatal closure, callose deposition and production of antimicrobial compounds [19]. However, it is unclear if or how Kac affects these defense pathways during fungal invasion of plant cells.

Southern corn rust (SCR) caused by *Puccinia polysora* (*P. polysora*), which causes severe yield losses and reduces nutritional quality, is becoming a major disease in maize production regions around the world [20, 21]. In recent years, this disease has become prevalent and difficult to control due to the dispersal and long-distance transmission of *P. polysora* urediniospores by wind currents [20, 21]. Identification and application of resistant genes for resistance to SCR in maize is limited by the rapid evolution of *P. polysora* and the emergence of multiple physiological races of *P. polysora* [22]. In addition, since *P. polysora* is an obligate biotrophic parasite that is difficult to culture on artificial media, thus increasing the difficulty of the study process on SCR. As a reversible PTM, Kac is regulated by the flux of acetyl-CoA and NAD^+^ in cells [5]. And acetyl-CoA and NAD^+^ are intermediate metabolites essential for cellular metabolisms, and their concentrations are regulated by various enzymes during cellular metabolisms [5, 23, 24]. Previous study has shown that the cellular metabolism of maize is significantly differentially regulated during fungi infection [25]. Increasing evidence that the TIR domain of the TNLs receptors in plant possess NADase activities and may be a signaling module for the induction of cell death and immune response [26, 27]. The Kac study on maize treated with fungal effector molecule HCT shows that the activity of plant encoded endogenous enzymes (histone deacetylases) can be modulated to alter non-histones acetylation during immune response [28]. These reports suggest that plants also use altered intracellular protein acetylation to cope with microbial invasion.

The blast of SCR disease depends on the successful germination, penetration, establishment and spread of *P. polysora* urediniospores, which is a dynamically regulated process. To explore the potential roles of Kac in maize infection with *P. polysora*, the isolated predominant physiological race PP.CN1.0 of *P. polysora* [29] was used to detect changes of protein acetylation during infection. To gain insight into Kac dynamics during maize and fungal interactions, we employed an optimized global Kac detection method [30] to investigate acetylproteomics (acetylome) triggered by *P. polysora* physiological race PP.CN1.0 at 0 h, 12 h, 24 h, 48 h and 72 h in SCR-resistant maize CML496 and -susceptible maize Lx9801. We found that nearly 96% of the Kac sites and proteins in this study were newly identified compared to the published acetylome datasets [28, 31]. And each time point has over 1800 Kac proteins and 2600 Kac sites. Bioinformatics analysis showed that the Kac targeted proteins are enriched in defense response pathways except for the basic biological pathways described in previously studies. Moreover, SCR pathogenic fungi altered not only histone acetylation but also acetylation of proteins involved in immune response, energy budget, proteostasis, ROS scavenging and cell wall remodeling. And the Kac levels of some Kac targeted immune relevant proteins presented reverse patterns in SCR-resistant and -susceptible maize. Thus, the data set presented here provides a comprehensive understanding of dynamic lysine acetylation in maize, improves our knowledge about plant-pathogen interactions in plants and may be used as a resource for SCR-resistant maize breeding.

## Material and methods

### Plant materials and infection with *P. polysora* in the growth chamber

Five SCR resistant (CML496) and -susceptible (Lx9801) maize plants were grown in individual pot filled with standard soil. The plants were placed in a growth chamber with photocycle of 16 h with light at 20 °C and 8 h without light at 18 °C and a relative humidity of around 60%. After the fifth true leaf was fully expanded, a freshly made *P. polysora* spore suspension (a concentration of 1 × 10^6^ spores mL^− 1^ contained a final concentration of 0.02% Tween-20) of the physiological race PP.CN1.0 isolated from leaves of -susceptible maize line Lx9801 [29] was sprayed uniformly into the leaves of the whole plants. The third and fourth leaves of five maize plants were harvested together as biological samples after 0 h, 12 h, 24 h, 48 h and 72 h of spraying, respectively. Three biological replicates were taken at each time point. The harvested maize material was quickly frozen in liquid nitrogen and stored in a refrigerator at − 80 °C for the next experiment.

### Protein extraction and in-solution digestion

Harvested samples were ground into fine powder using liquid nitrogen, and then transferred into a new 15 mL ice-cold centrifuge tube. The total protein of the biological samples at each time point was extracted using the phenol-based method as described previously [32]. The protein concentrations were measured using the Pierce™ 660 nm Protein Assay (Thermo Scientific, #22660) following standard protocols. The precipitated proteins were digested and desalted as described previously [30]. The desalted peptides were lyophilized with lyophilizer (SCIENTZ-10YG/A).

### Enrichment of the acetylated peptides

Three mg of lyophilized peptides from each sample were dissolved in NETN buffer (100 mM NaCl, 1 mM EDTA, 50 mM Tris-HCl, 0.5% NP-40, pH 8.0), the peptide concentrations were measured using the Pierce™ BCA Protein Assay (Thermo Scientific, #23225) following standard protocols. The peptides were then incubated overnight at 4 °C with 20 μL PTM Biolabs Inc. (PTM-104) and 40 μL Cell signaling Tech Inc. (#13416) pre-washed anti-acetyl-lysine beads with gentle rotation [30]. The beads were washed four times with cold NETN buffer and three times with cold ddH_2_O. The bound peptides were then eluted from the beads using 0.5% TFA (Trifluoroacetic acid), and the eluted peptides were loaded onto pre-wet Pierce C18 Zip Tips (Thermo Scientific, 87,784) for desalting according to the manufacturer’s instructions. The resulting peptides were lyophilized with lyophilizer.

### LC-MS/MS analysis of total protein using data-independent acquisition (DIA) approach

#### RPLC analysis

Total protein digested peptides from each sample were separated on an 1100 HPLC System (Agilent) using Agilent Zorbax Extend RP column (5 μm, 150 mm × 2.1 mm). Mobile phases A (2% acetone nitrile (ACN) in HPLC water) and B (90% ACN in HPLC water) were used for RP gradient at a fluent flow rate of 250 μL min^− 1^. The solvent gradient was set as follows: 2% solvent B for 0–10 min, 2–5% B for 10–10.01 min, 5–20% B for 10.01–37 min, 20–40% B for 37–48 min, 40–90% B for 48–48.01 min, 90% B for 48.01–58 min, 90–2% B for 58–58.01, 2% B for 58.01–63 min. Fractions of each sample were collected in centrifuge tubes labeled 1 to 10 at one-minute intervals starting at 10 min. Fractions were collected in cycles from 1 to 10 until the end of the separation gradient. The fractionation peptides were lyophilized for MS analysis.

#### Mass spectrometry analysis

All fractionation peptides of each sample were dissolved in 0.1% formic acid (FA) and analyzed by Easy-nLC 1200 system coupled with a Q Exactive HF mass spectometer (Thermo, Bremen, Germany) at a flow rate of 300 nL min^− 1^ with a linear gradient increasing solvent B (0.1% FA in 80% ACN) over 60 min from 8 to 25%, 25 to 45% for 79 to 80 min, and 45 to 100% for 80 to 90 min [30]. For data-dependent acquisition (DDA), full MS scans were acquired in the mass range of 350–1250 m z^− 1^ with a mass resolution of 60,000 and the automatic gain controls (AGC) target value was set at 4e5, and the top 20 most intense peaks in MS1 were fragmented using higher-energy collisional dissociation (HCD) with a collision energy of 35. The tandem MS (MS/MS) spectra were obtained with a resolution of 15,000 with an AGC target of 5e4 and a maximum injection time of 60 ms. The dynamic exclusion was set to 45 s and runs at positive mode. For DIA, full MS scans were acquired in the mass range of 350–1250 m z^− 1^ with a mass resolution of 60,000 and the AGC target value was set at 4e5. 32 acquisition windows in MS were fragmented using HCD with collision energy of 35 and each acquisition window has 26 m z^− 1^. MS/MS spectra were obtained with a resolution of 30,000 with an AGC target of 1e5 and a max injection time of 100 ms and run at positive mode.

### LC-MS/MS analysis of acetylated peptides using DDA method

The acetylated peptides point were analyzed at each time using tandem mass spectrometry as described previously [30]. Briefly, acetyl-lysine peptides were reconstituted in 0.1% FA and analyzed on an online nanoAcquity ultraperformance LC (Waters; USA) coupled with an Orbitrap Fusion Tribrid mass spectrometer (Thermo, Watham, MA, USA). Nanospray was controlled by a PicoView Nanospray Source (PV550; USA) at a spray voltage of 1.9 kV. The peptides were trapped by a 2G-V/MT Trap symmetry C18 column (5 μm particles, 180 μm ID × 20 mm length) at the flow rate of 5 μL min^− 1^ for 3 minutes and separated on a BEH130 C18 analytical column (1.7 μm particles, 100 μm ID × 250 mm length) at 250 nL min^− 1^ with 120 min linear gradient of 3 ~ 85% ACN in 0.1% fluoroacetate. DDA acquisition was performed following a full MS scan by Orbitrap at a resolution of 120,000 over the m/z range of 350–1500 and the top 20 most intense peaks in MS were fragmented with HCD at collision energy of 32. The max injection time was set as 50 ms and the target values of AGC were set up to 200,000 for Orbitrap MS. The max injection time was set as 120 ms, and the target values of AGC were set up to 100,000 for ion-trap MS/MS detection at a resolution of 15,000. The fragmentations of the selected multiply charge ions were achieved using helium gas and argon at a normalized collision energy of 35% for HCD. The dynamic exclusion was set for 60 s and run at positive mode.

### Database search of total proteins quantified by DIA

To gain a comprehensive understanding of protein changes during *P. polysora* infection, the protein databases of B73 and Mo17 from Uniprot (https://www.uniprot.org/) and the Zea_mays.AGPv3.22 from MaizeGDB (https://download.maizegdb.org/) were merged by eliminating redundant proteins based on their sequences. DDA library generated by Spectronaut™ 15.0 software (Biogenosys, Schlieren, Switzerland) based on the following parameters. The raw data acquired by DDA were thoroughly against the merged database (166,083 entries, the above merged database) concatenated with reverse decoy database and protein sequences of common contaminants, the enzyme specificity was set to trypsin with a maximum miscleavage of 2, carbamidomethylation of Cys was set a fixed modification, oxidation of Met was set as variable modification. The threshold of false discovery rate (FDR) was set at 0.01 for proteins, peptides and peptide-spectral matches (PSMs). All other search parameters were set to default settings of Spectronaut Pulsar. For quantification of DIA data, all parameters were set consistent with the above except that the threshold of FDR for proteins, peptides and PSMs was set to 0.05, the normalization strategy was set to local normalization. The quantity MS-level was set at MS2.

### Database search for Kac peptides

To gain a comprehensive understanding of the acetylome of maize under *P. polysora* infection, two database search strategies were performed, one for identification and one for quantification. For identification, the raw data generated by LC-MS/MS were searched against the merged database (details as above) with a concatenated decoy database with contaminants using the Open-pFind search algorithm of the pFind 3.1.6 integrated into pFind Studio (http://pfind.ict.ac.cn/software/pFind/). For quantification, three search algorithms including MS Amanda, Sequest HT and Mascot were applied for spectrum selection, the resulting data were combined using the multi-consensus feature of percolator with consistent FDR in Proteome Discoverer 2.4.0 software (Thermo Fisher Scientific Inc.). For all search tools, the precursor mass tolerance was set to 20 ppm. The fragment mass tolerance of pFind and PD were set to 20 ppm and 0.8 Da, respectively. The carbamidomethylation of Cys was set as a fixed modification while deamidation of Asn/Gln, oxidation of Met and acetylation of Lys were set as variable modifications. The enzyme specificity was limited to trypsin with the maximum number of missed cleavages of 4, and the maximum number of modifications per peptide set to 5. The threshold of FDR was set at 0.05 for proteins, peptides and PSMs. All other parameters were set to the default values for each search algorithm and software.

### Histone immunoblot analysis

The ground fine powder of each time point was mixed with extraction buffer containing 100 mM Tris, pH 8.0, 1.5 M β-mercaptoethanol, 4% SDS, 15% glycerol, 2 mM EDTA, 0.005% bromophenol blue, 2 μM/mL Trichostatin A (TSA) and vortexed for 30 s. The mixed solution was boiled for 5 min at 99 °C and vortexed 30 s. The supernatants were transferred into a new collection tube after centrifugation at 15,000 g for 10 min at RT. Proteins were loaded into 12% sodium dodecylsulfate-polyacryamide gel electrophoresis (SDS-PAGE) for protein separation and transferred to a polyvinylidene fluoride (PVDF) membrane, and probed using anti-H3 (ab1791) and anti-H3K18ac (ab1191) from Abcam, anti-H3K23ac (#07–355), anti-H3AC (#06–599), anti-H4K5ac (#07–327), anti-H4K8ac (#07–328), anti-H4K12ac (#07–595) and anti-H4K16ac (#07–329) from Millipore at a dilution of 1:2000 (v/v) in 5% skim-milk TBST (10 mM Tris-HCl, pH 75, 150 mM NaCl, and 0.1% Tween 20) for 1 h at RT after incubation with blocking buffer for 1 h at RT. The membranes were washed three times with TBST for 5 min each. Next, membranes were incubated with secondary antibody (AS014, ABclonal, Inc) in 5% skim-milk-TBST for 1 h at RT. Signals in membranes were detected using the BLT Gel View 6000 Plus machine (Biolight Biotec Co., Ltd.).

### Bioinformatics analysis

Gene Ontology (GO) annotations (GOA) for quantified proteins and identified or quantified acetylated proteins were derived from the GOA database (https://www.ebi.ac.uk/GOA/). The subcellular localizations of the Kac proteins were predicated using BUSCA (http://busca.biocomp.unibo.it/). Sequence motifs were analyzed by pLogo [33]. KEGG pathway was annotated using Kyoto Encyclopedia of Genes and Genomes (KEGG) [34]. Gene set enrichment analysis and KEGG pathway enrichment were performed using the clusterProfiler package in R (4.0.5). For all enrichment analysis of Kac proteins were evaluated using two-tailed Fisher’s exact test, corrections for multiple hypothesis testing were implemented using the standard FDR control method proposed by Benjamin and Hochberg. The annotation categories with a corrected *p*-value ≤0.01 and FDR ≤ 0.05 were considered significant. The intermediate data processing was performed using in-house developed Perl scripts and shell scripts. All the tile plots, boxplots, and bar plots were generated using the ggplot2 package of R, heatmaps were generated using the pheatmap package of R.

### Experimental design and statistical rationale

Three biological replicates were performed at each time point of 0 h, 12 h, 24 h, 48 h and 72 h in SCR-resistant and -susceptible maize. The samples used for protein quantification and acetylated protein quantification are from the same biological replicate. All samples used for protein quantification and acetylated protein quantification were performed following the experimental procedures described above. After LC-MS/MS analysis and database search, the results of protein quantification experiments were summarized into one matrix with FDR was set to 0.01 for protein and peptide levels, and the results of acetylated protein quantification experiments were summarized into another matrix with FDR was set to 0.05 for protein and peptide levels. Global normalization of each matrix was performed using Equalize Medians parameters, hypothesis testing was conducted using moderated t-test, and multiple comparisons were performed using the Benjamini-Hochberg adjustment method. Differentially expressed levels of acetylated proteins were determined after protein level normalization by fold changes > 1.5 with t-test *p*-value < 0.05, reflecting a shifting of average intensity at each time point in three replicates for SRC-resistant and -susceptible maize.

## Results

### Quantitative profiling of proteome and acetylome of maize with *P. polysora* infection

**Fig. 1:**
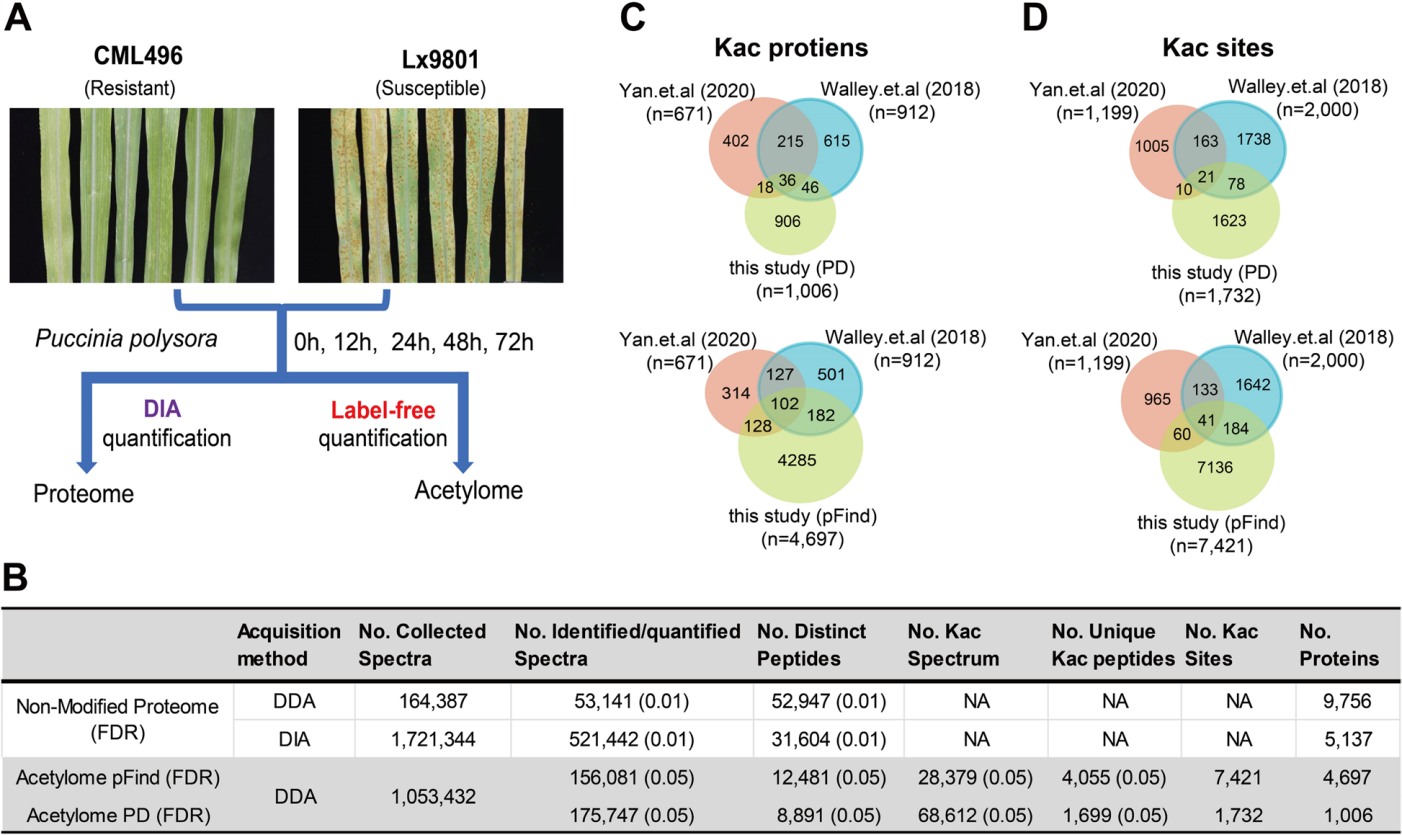
Overview of proteome and acetylome profiling of maize. **A** Strategies for data-independent acquisition (DIA) quantitative proteomics and data-dependent acquisition (DDA) label-free quantitative acetylome analysis of SCR-resistant and -susceptible maize plants treated with *P. polysora* for 0 h, 12 h, 24 h, 48 h and 72 h. **B** Summary of spectra, peptides, acetylated peptides and identified proteins of samples used in this study. **C** and **D** Comparison of the lysine acetylated proteins (**C**) and sites (**D**) quantified and identified in this study with the published datasets (Walley et al., 2018; Yan et al., 2020). FDR, false discovery rate

MS-based DIA and DDA label-free quantitative methods were used to quantify the changes in protein abundance and specific lysine residues acetylation in SCR-resistant maize CML496 and -susceptible maize Lx9801 infected with *P. polysora* at 0 h, 12 h, 24 h, 48 h and 72 h (Fig. 1A). 164,387 spectra were collected by DDA acquisition method for spectral library preparation of DIA quantification, of which 53,141 spectra with FDR ≤ 0.01 were identified to 9756 proteins. 1,721,344 spectra obtained by DIA method were quantified into 5137 proteins with FDR ≤ 0.01 on the basis of DDA spectral library (Fig. 1B, Table S1). In parallel, 28,379 and 68,612 Kac spectra with FDR ≤ 0.05 were identified and quantified from 1,053,432 collected spectra of enriched Kac peptides and mapped to 4055 and 1699 unique Kac peptides using pFind and PD software, respectively (Fig. 1B, Table S2 and S3). Meanwhile, 7412 Kac sites of 4697 proteins were found in 4055 uniquely identified Kac peptides, and 1732 Kac sites of 1006 proteins were found in 1699 uniquely quantified Kac peptides, respectively (Fig. 1B, Table S3).

To evaluate this global acetylome data, we compared the identified and quantified Kac sites and proteins with two recently published acetylomes [28, 31], and found 1680 novel Kac sites in quantified dataset and 7128 novel Kac sites in identified dataset (Fig. 1C, D). However, the number of shared Kac sites and proteins of 3 datasets is quiet low. The possibility is that each study is under sampling the acetylome which gives to the low overlap. Because different maize cultivars, growth stages and conditions were used in these studies for Kac analysis. For instance, the overlaps of the Kac sites and Kac proteins quantified in the studies of Yan et al. and Walley et al. are only 8.65% (173/2000) and 25.10% (229/912) (Fig. 1C, D) even the same cultivar maize B73 and protein reference database used in their studies [28, 31]. To know the common Kac proteins in maize defense, we analyzed the shared Kac proteins (284) identified by Walley et al. [28] and this work and found Kac proteins are involved in the assembly of complexes and regulation of protein activity, such as carbon metabolism, nucleosome and chromatin assembly, generation of precursor metabolites and energy, protein folding and abiotic stimulus response etc. (Fig. S1).

### A catalog of identified Kac proteins in maize infected with *P. polysora*

**Fig. 2:**
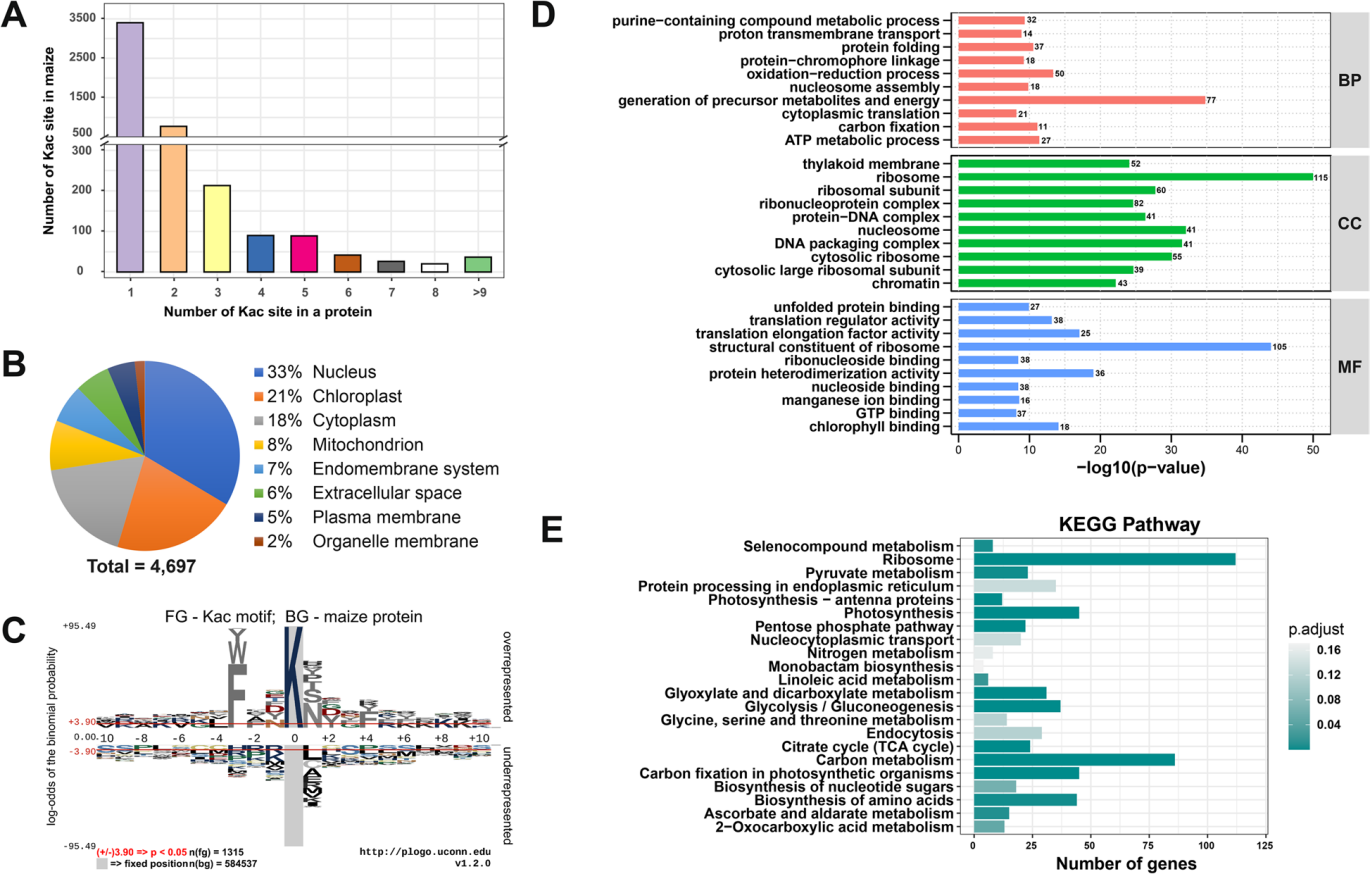
Characteristics of Kac proteins identified in maize infected with *P. polysora*. **A** Distribution of Kac sites per identified protein. **B** Subcellular localization of identified Kac proteins. **C** Sequence logo of all identified Kac sites with all proteins as background population (generated with pLogo). **D** Tops10 GO (gene ontology) enrichment analysis of the identified Kac genes. The numbers on the right side of the bar indicate the number of Kac genes enriched in item. BP, biological process; CC, cellular component; MF, molecular function. **E** KEGG pathway enrichment analysis of all identified Kac proteins

To understand the global landscape of Kac in plant-pathogen interactions, the identified Kac dataset was further analyzed. We found the number of Kac sites per protein was basically one or two, and also 27% of proteins had more than 2 Kac sites (Fig. 2A, Table S3). In eukaryotic cells, diverse cellular activities are compartmentalized in a spatially and temporally controlled manner in various membrane-bound organelles [35]. Analyzed the subcellular localization of Kac proteins utilizing the BUSCA (Bologna Unified Subcellular Component Annotator) [36]. We found that Kac proteins account for 33% in the nucleus and over 35% in chloroplast and cytoplasm (Fig. 2B, Table S2). And nearly similar number of Kac proteins were localized in the mitochondrion, endomembrane system, extracellular space and plasma membrane (Fig. 2B, Table S2). Further analyzed the 15 amino acid flanking sequences at the N- and C-terminal of the Kac site in different organelles, the results showed that polar neutral amino acid, including Asn (N), Ser (S), Thr (T), Pro (P), Tyr (Y) and Gly (G), were overrepresented at the + 1 position (the 1st residue at the C-terminal side of the Kac), whereas non-polar hydrophobic amino acid, including Leu (L), Cys (C), Ala (A) and Phe (F), and the positively charged amino acid Arg (R) and Lys (K), were underrepresented at + 1 (Fig. S2). Moreover, non-polar hydrophobic aromatic amino acid residues including Phe (F) and Trp (W) and the polar neutral amino acid residues Tyr (Y) were overrepresented at the − 3 position in all organelles (Fig. 2C, Fig. S2).

Enriched Gene Ontology (GO) terms showed that the Kac proteins were mainly concentrated in macromolecular complexes and organelle membranes, regulating protein binding ability and enzyme activity involved in membrane transport, protein folding, complex assembly and basic metabolic process (Fig. 2D, Fig. S3). The results of KEGG pathway analysis exhibited that in addition to the common terms such as ribosome, pyruvate metabolism, photosynthesis, pentose phosphate pathway, citrate cycle (TCA cycle) and carbon metabolism were enriched in previous Kac studies [30, 31] and shared in [28] (Fig. S1), defense-associated pathways were particularly enriched in the pathogen-infected maize, such as the ascorbate metabolism, oxocarboxylic acid metabolism, monobactam biosynthesis, linoleic acid metabolism and endocytosis (Fig. 2E). These results suggest that Kac may alter the activity of metabolic components and endocytosis-associated proteins during pathogen invasion and regulate metabolic fluxes at crucial steps of the pathway.

### Landscapes of acetylome in SCR-resistant and -susceptible maize induced by *P. polysora*

**Fig. 3:**
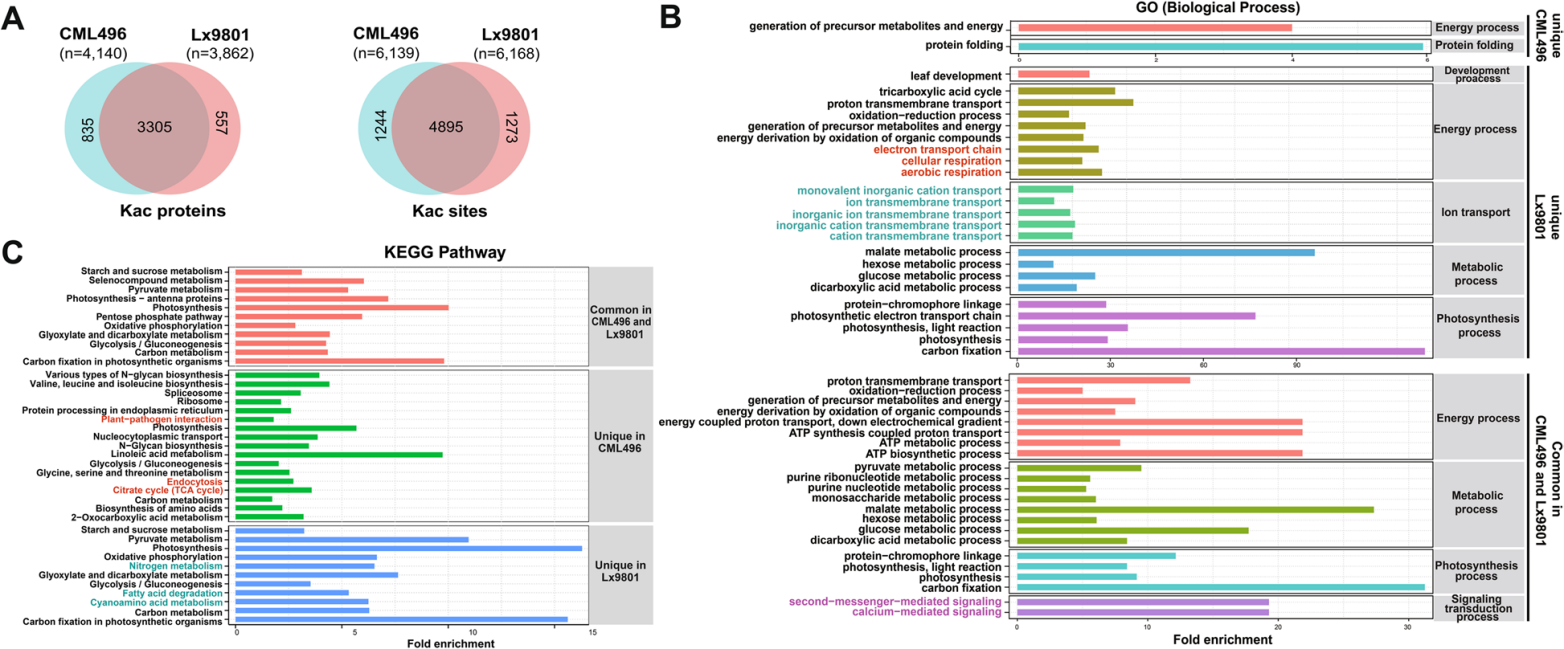
Differences in Kac proteins of SCR-resistant and -susceptible maize*.* **A** Comparison of Kac proteins and sites in CML496 and Lx9801. **B** Biological process enrichment analysis of all identified common and specific Kac proteins in CML496 and Lx9801. **C** KEGG pathway enrichment analysis of all identified common and specific Kac proteins in CML496 and Lx9801

To know the difference in acetylation between SCR-resistant and -susceptible maize infected with *P. polysora*, the acetylomes of CML496 and Lx9801 were compared. Before comparing the Kac proteins and sites in CML496 and Lx9801, we examined the number of Kac sites and proteins at each time point, and the results showed that distribution of Kac sites and proteins at each time point was close to the same level (Fig. S4). The overlap of Kac proteins and sites in CML496 and Lx9801 is more than 80%, even though about 20% Kac sites and 14% ~ 20% Kac proteins are specific to each other, indicating that the detection of the Kac proteins and sites is reproducible (Fig. 3A). GO Analysis of common and specific Kac proteins of CML496 and Lx9801 showed that the common Kac proteins of CML496 and Lx9801 concentrated in basic metabolic pathways, signaling transduction and energy process and were involved in the regulation of metabolic enzyme activity and transmembrane transporter activity. Specific Kac proteins in CML496 aggregated in protein folding and were involved in nucleoside binding and protein translation, while the specific Kac proteins in Lx9801 gathered in electron transport and transmembrane transport and were involved in regulation of enzyme activities associated redox reactions (Fig. 3B, Fig. S5, S6). Further analyzed the KEGG enrichment of common and specific Kac proteins in CML496 and Lx9801 revealed that the plant-pathogen interaction and endocytosis pathways were uniquely enriched in CML496 (Fig. 3C). These results suggest that proteins in immune-associated processes and pathways are differently regulated by Kac.

### *P. Polysora* infection regulates cellular processes by altering Kac levels of histone and non-histone

**Fig. 4:**
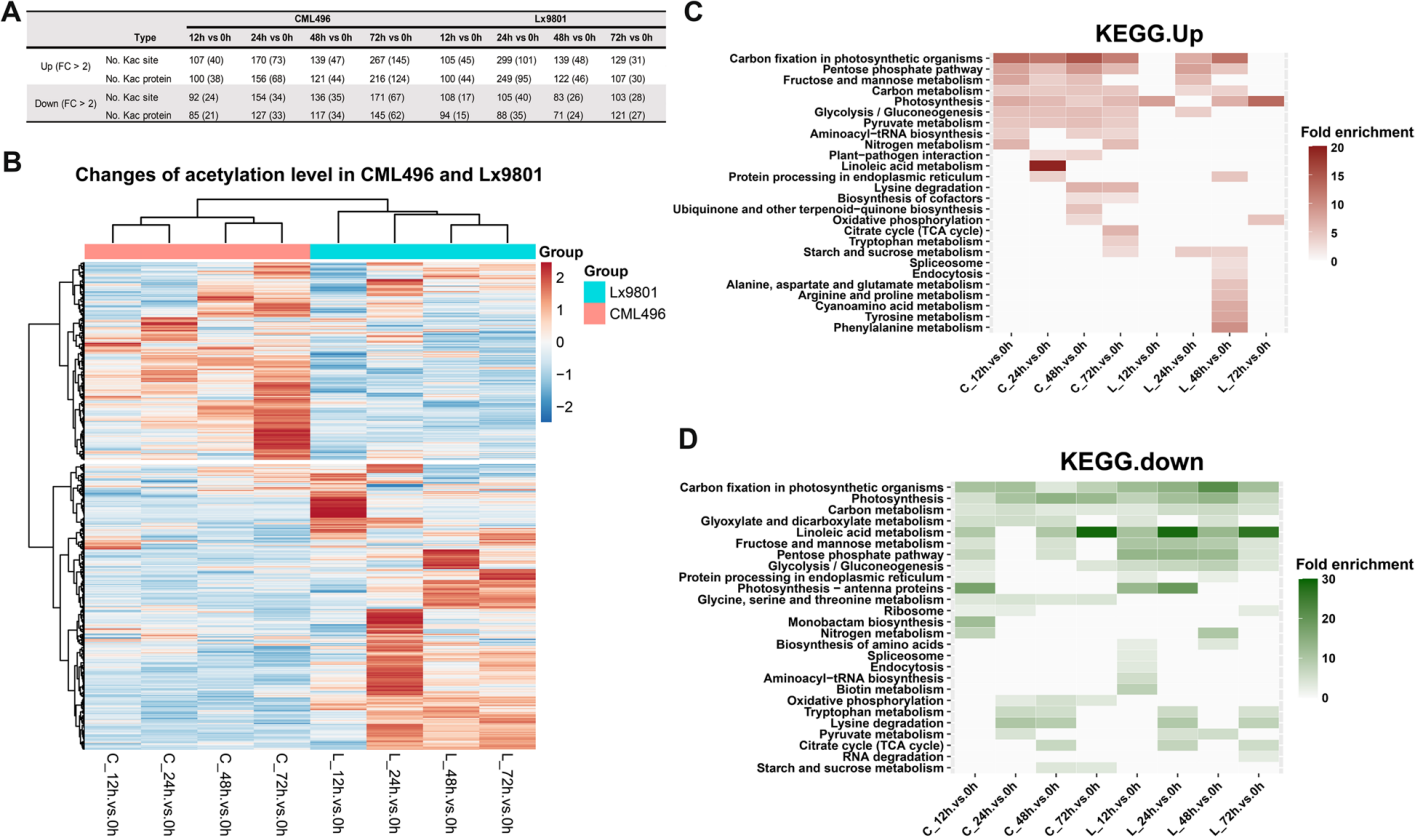
Differential regulation of Kac in SCR-resistant and -susceptible maize*.* **A** Summary of the differentially regulated Kac proteins and sites in CML496 and Lx9801 during *P. polysora* invasion. **B** Heatmap showing changes in differential Kac proteins and sites of CML496 and Lx9801 with *P. polysora* infection. **C** and **D** KEGG pathway enrichment analysis of Kac protein up-regulation (**c**) and down-regulation (**d**) after CML496 and Lx9801 infected with *P. polysora*. FC, fold change. C, CML496; L, Lx9801

Lysine acetylation is believed to provide a mechanistic link between metabolic state and cellular signaling [37]. To know the changes of whole protein Kac levels in maize during fungal infection, we examined the acetylation levels of CML496 and Lx9801 maize infected with *P. polysora* at 0 h, 12 h, 24 h, 48 h and 72 h according to the pathological process of *P. polysora* [38]. After normalization with non-enriched proteins, acetylation levels at 12 h, 24 h, 48 h and 72 h were compared with 0 h, indicating that changes in acetylation levels were rarely associated with protein abundance and were mainly concentrated in D and F quadrants in the nine-box quadrant of proteome versus acetylome (Fig. S7). For Kac proteins that were not detected at the whole protein level, changes in their protein levels were ignored. Summarizing the acetylation fold changes of Kac sites and proteins, we found that acetylation of CML496 were mainly up-regulated and down-regulated at 72 h, while acetylation of Lx9801 were principally at 24 h (Fig. 4A, B). Interestingly, acetylation of CML496 and Lx9801 showed almost completely reverse changes (Fig. 4B). Results of KEGG pathway enrichment analysis of differentially regulated Kac proteins (DKPs) with a fold change > 1.5 (Kac proteins normalized by protein abundance in quadrants A, C, D, F, G and I (Fig. S7)) showed that acetylation of proteins involved in photosynthesis, carbon metabolism, fructose and mannose metabolism, pentose phosphate pathway and glycolysis/gluconeogenesis in CML496 and Lx9801 were differentially regulated at 12 h, 24 h, 48 h and 72 h of PP.CN1.0 infection, suggesting that these basal metabolic processes are regulated by Kac during pathogenic infestation (Fig. 4C, D). Moreover, acetylation of proteins involved in plant-pathogen interactions were specifically up-regulated at 24 h and 48 h in CML496 infected with PP.CN1.0 (Fig. 4C). Further compared GO analysis of DKPs in CML496 and Lx9801 displayed proteins involved in protein folding and correction, energy metabolism and proton transport were specifically enriched in DKPs that were up-regulated at 24 h and 72 h in CML496 (Fig. S8A). Proteins involved in electron transport and complex assembly and disassembly were particularly gathered at DKPs in Lx9801 at 12 h and 24 h (Fig. S8). Proteins that response to jasmonic acid were specifically enriched in DKPs that were down-regulated at 24 h in Lx9801 (Fig. S8B).

**Fig. 5:**
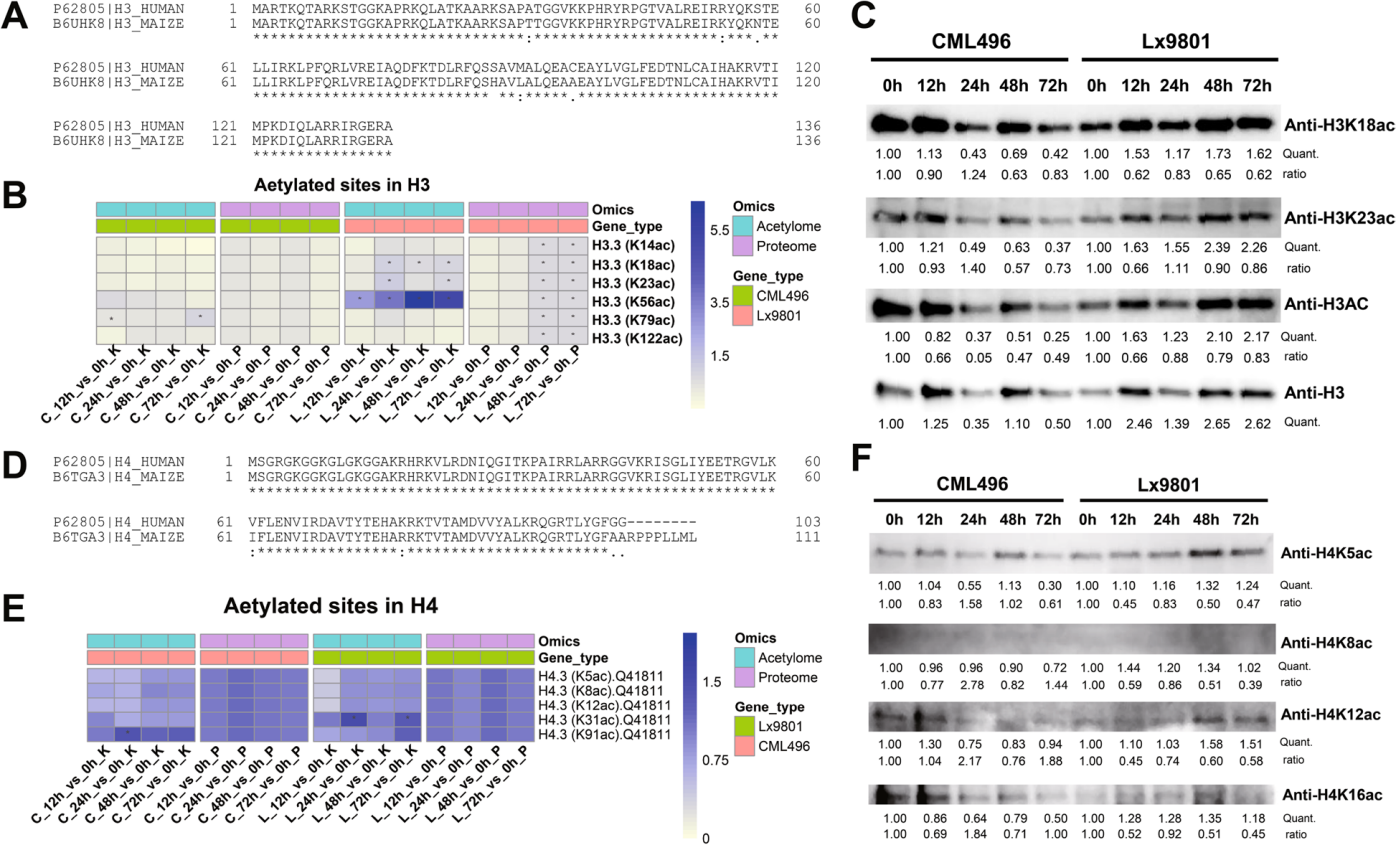
Validation of the acetylome quantified in maize with *P. polysora* infection*.* **A** and **D** Alignment of H3 (**A**) and H4 (**D**) sequences of human and maize. Asterisks (*) indicate conserved amino acid sites, dots (·) indicate relatively conserved amino acids, colons (:) indicates slightly conserved amino acids. **B** and **E** Quantification of specific Kac sites in H3 (**B**) and H4 (**E**) of proteome and acetylome. Asterisks (*) indicate fold change > 1.5. C, CML496; L, Lx9801. **C** Western blot showing the levels of H3 protein and acetylation levels of Lys18 (K18ac), Lys23 (K23ac) and pan-acetylation (H3AC) of H3. The relative intensities of H3 and H3 specific Kac sites at 0 h treatment was set to 1.00. **F** Western blot showing acetylation levels of Lys5 (K5ac), Lys8 (K8ac) and Lys12 (K12ac) of H4. The relative intensity of the H4 specific Kac sites at 0 h treatment was set to 1.00. Quant. indicates the intensity measured by ImageJ. Ratio Indicate the intensity was normalized to anti-H3

To validate the acetylome data, we verified the changes of acetylation using commercial antibodies that recognize specific Kac sites of histone H3 and H4. Before verifying acetylation levels of H3 and H4 Kac sites, we analyzed the conservativeness of H3, H4 and their variants (Fig. S9), since almost all commercial antibodies were generated from human protein sequences. The alignment results exhibited that the sequences of human H3 and H4 used in commercial antibodies are extremely similar to maize H3 and H4 (Fig. 5A, D). The MS data of H3 specific Kac sites normalized to H3 protein abundance displayed that acetylation level at Lys14 of H3 in CML496 and Lx9801 were almost unchanged, but acetylation levels at Lys18 and Lys23 of H3 in CML496 and Lx9801 were slightly decrease, which was consistent with western blotting results (Fig. 5B, C). The MS data of acetylation level at Lys56 of H3 were significantly elevated in Lx9801 (Fig. 5B). However, since no Lys56 Kac specific antibodies were available, we measured the total acetylation levels in H3 with H3 pan-acetyl antibodies, which showed reduced acetylation levels in H3 (Fig. 5C), suggesting that acetylation levels may be reduced at other sites in H3 during pathogen infection but were not detected in this study. MS data of H4 acetylation levels showed relatively low variation during *P. polysora* infection (Fig. 5E), which was consistent with the results of H4 specific Kac sites detected by western blotting (Fig. 5F). A previous report showed that *C. carbonum* secreted HCT, an inhibitor of KDACs, facilitated elevated levels of tetra-acetylation of Lys 5, Lys8, Lys12 and Lys16 in H4 [28]. Thus, we detected acetylation levels of Lys5, Lys8, Lys12 and Lys16 in H4 by western blotting using Kac site specific antibodies. Due to the lack of H4 antibodies, we normalized the signal intensity of H4 specific Kac sites to H3 protein levels. The results showed that the acetylation levels of these H4 Kac sites were slightly up-regulated in CML496 and down-regulated in Lx9801 (Fig. 5F). It suggests that *P. polysora* may secrete effectors encoding KATs inhibitors or KDACs to prevent histone acetylation in SCR-susceptible maize, and thereby regulating the expression of immune-related proteins.

### Immune-associated proteins in maize were dynamically regulated by Kac during *P. polysora* invasion

From pathogen recognition to colonization of plant cells, PTI and ETI induce a diverse array of immune responses, including ROS burst, MAPK cascade activation, JA biosynthesis and transcriptional reprogramming [19]. Increasing evidence that Kac plays a crucial role in plant-pathogen interactions [8, 28, 39]. Analysis of quantitative acetylome data reveals potential functions of Kac in the regulation of immune responses.

#### Defense-relevant proteins

**Fig. 6:**
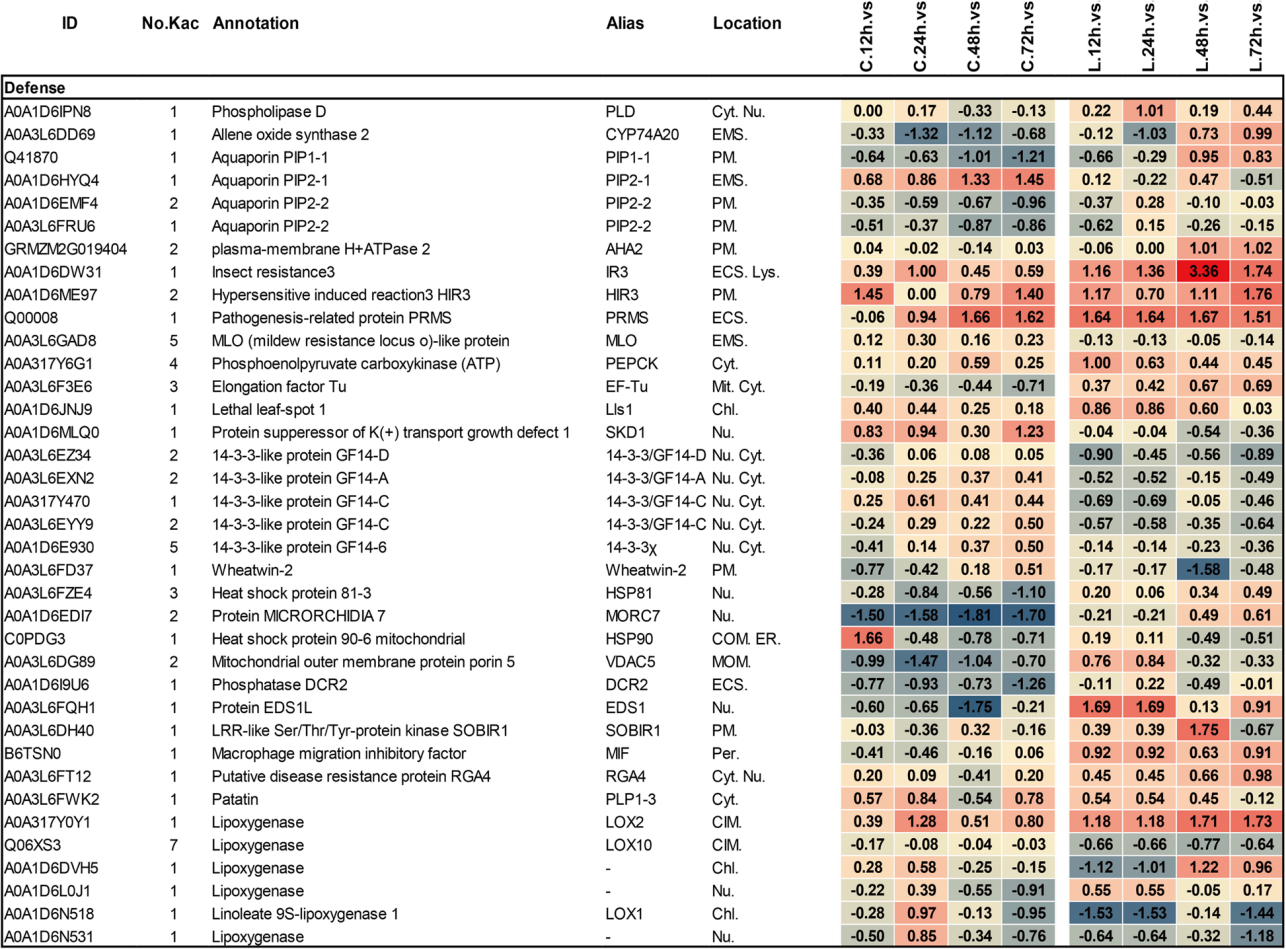
Heat map of differentially acetylated proteins involved in plant defense found in CML496 and Lx9801 infected with *P. polysora.* Abbreviations: Cyt, cytoplasm; Nu, nucleus; Chl, chloroplast; Mit, mitochondrion; Lys, lysosome; PM, plasma membrane; Per, peroxisome; ECS, extracellular space; EMS, endomembrane system; COM, chloroplast outer membrane; CIM, chloroplast inner membrane; MOM, mitochondrial outer membrane. C, CML496; L, Lx9801

We found serval defense response proteins were acetylated in this study. For example, SOBIR1, a leucine-rich repeat (LRR)-like protein kinase involved in NLP-triggered immunity [40], showed decreased acetylation in CML496 compared with Lx9801 at 12 h, 24 h and 48 h of PP.CN1.0 infection (Fig. 6). EDS1, is essential for all TNL-mediated resistance responses and interacts with PAD4 or SAG101 modules in response to different pathogen attacks [41], its acetylation was down-regulated in CML496 but up-regulated in Lx9801 after PP.CN1.0 infection (Fig. 6). HIR3, a PM localized hypersensitivity-induced response protein involved in cell death via EDS1 and SA-dependent pathways to drive basal resistance [42], its acetylation were differentially up-regulated during CML496 and Lx9801 infected with PP.CN1.0 (Fig. 6). RGA4, a NB-LRR disease resistance protein that interacts with RGA5 to confer disease resistance in plant [43–45], its acetylation was up-regulated in Lx9801 compared to CML496 after infection with PP.CN1.0 (Fig. 6). Lls1, a protein involved in cell death and disease tolerance [46], showed increased acetylation in both CML496 and Lx9801 after PP.CN1.0 infection (Fig. 6,). HSP81 and HSP90, proteins that cooperate with chaperones to regulate plant defense [47], were down-regulated in its acetylation in CML496 but slightly up-regulated in Lx9801, except for the HSP90 at 12 h of PP.CN1.0 infection (Fig. 6). Elongation factor Tu (EF-Tu), a protein involved in extracellular signalling perception [48], showed a slightly decrease in its acetylation in CML496 but an increase in Lx9801 during PP.CN1.0 invasion (Fig. 6). MORC7, an epigenetic regulator involved in transcription and DNA damage responses [49], was significantly down-regulated in acetylation in CML496, while little change was observed in Lx9801 under PP.CN1.0 invasion (Fig. 6). 14–3-3 proteins (14–3-3 s), are a family of ubiquitously expressed adaptor proteins which bound directly to RPM1-interacting protein4 (RIN4) and PM H^+^-ATPase AHA2 via GENERAL CONTROL NONREPRESSIBLE 4 (GCN4) to modulate stomatal aperture for control pathogen invasion [50, 51], and its acetylation of CML496 was slightly up-regulated at 24 h, 48 h and 72 h but down-regulated in Lx9801 during PP.CN1.0 infection (Fig. 6). Moreover, several additional immune proteins, including insect resistance 3 protein IR3, mildew resistant locus O-like protein MLO, wheatwin-2, patatin and macrophage migration inhibitory factor MIF, were also differentially regulated by Kac following PP.CN1.0 infection (Fig. 6). These findings suggest that acetylation may modulate the activity of immune relevant membrane proteins and intracellular immune proteins to defend against pathogen attack.

#### JA biosynthesis-associated proteins

Plants usually generate defense hormones jasmonic acid (JA), salic acid and ethylene to regulate defenses during stress responses [52]. In total, nine JA biosynthesis related proteins were differentially regulated by Kac in maize infected with *P. polysora*: two phospholipases PLD and PLP1–3, one allene oxide synthase 2 (CYP74A20), and six lipoxygenases (LOXs) (Fig. 6). PLD hydrolyzes membrane phospholipids to generate phosphatidic acid (PA) to promote JA biosynthesis [53]. CYP74A20 catalyzes the synthesis of allene oxide which is involved in the production of JA [54]. LOXs are enzymes that regulate the metabolism of polyunsaturated fatty acids, enabling the oxygenation of α-linolenic acid that promotes JA biosynthesis [55–57]. This suggests that Kac may affect the activity of JA biosynthesis metabolism enzymes in response to pathogen invasion.

#### Calcium-mediated signaling proteins

**Fig. 7:**
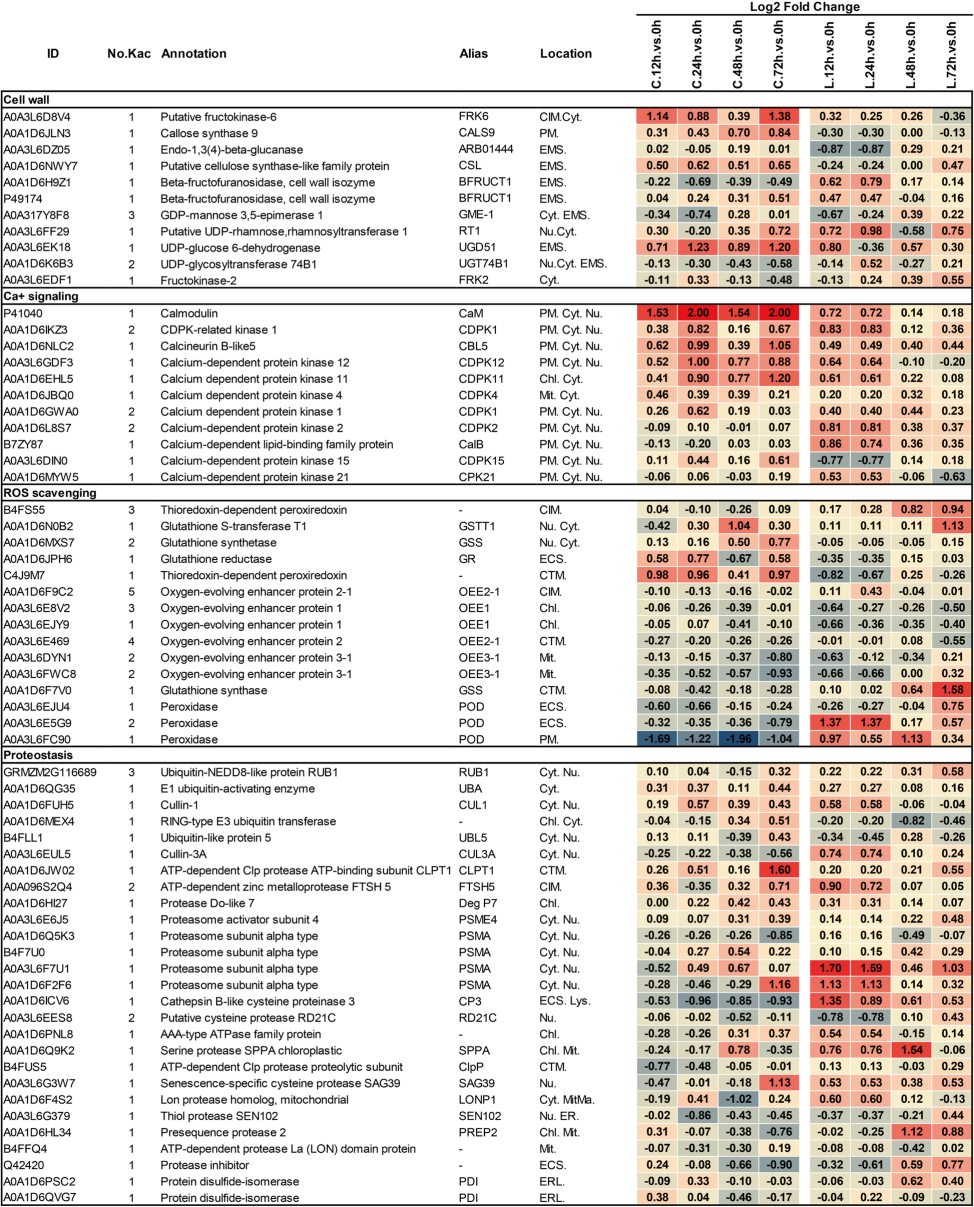
Heat map of differentially acetylated proteins involved in cell wall biosynthesis, Ca^2+^ signaling regulation, ROS scavenging, proteostasis found in CML496 and Lx9801 infected with *P. polysora.* Abbreviations: ER, endoplasmic reticulum; ERL, Endoplasmic reticulum lumen; CTM, chloroplast thylakoid membrane; MitMa, mitochondrion matrix; the rest is the same as Fig. 6

Calcium (Ca^2+^) is a second messenger for cell surface and intracellular receptors and mediates the signaling transduction involved in plant immune responses [58]. We found that acetylation of CDPKs quantified at all time points were elevated in CML496 except for CDPK2, CalB and CDPK21, while acetylation of all these three CDPKs were significantly increased at 12 h and 24 h in Lx9801 in presence of *P. polysora* (Fig. 7). In particular, calmodulin (CaM), a Ca^2+^ signaling molecule, binds and activates many target proteins, including Ca^2+^/CaM-dependent protein kinases (CaMK) family proteins and nucleotide-binding leucine-rich repeat receptors (NLRs), and plays an important role in plant immune defense [59, 60], we found its acetylation was increased significantly at each time point in CML496 but only slightly in Lx9801 after infection with PP.CN1.0 (Fig. 7).

#### Cell wall remodeling proteins

A major difference between plant cells and animal cells is that plants have cell walls. Pathogens destroy plant defenses by degrading the cell wall, in return, plant cells control their homeostasis to fortify the cell walls and thus defend themselves against pathogen attacks [17]. Eleven cell wall remodeling related proteins, including fructokinase-6 (FRK6), callose synthase 9 (CALS9), endo-1,3(4)-beta-glucanase (ARB01444), two isoforms of beta-fructofuranosidases (BFRUCT1), cellulose synthase-like protein (CSL), GDP-mannose 3,5-epimerase 1 (GME-1), UDP-glucose 6 dehydrogenase (UGD51), UDP-glycosyltransferase 74B1 (UG74B1), UDP-rhamnose: rhamnosyltransferase 1 (RT1) and fructokinase-2 (FRK2) [17, 61–63], were found differentially acetylated upon PP.CN1.0 invasion (Fig. 7). Among them, acetylation of FRK6, UGD51, CALS9 and CSL were elevated in CML496, of ARB01444, BFRUCT1 (P49174), RT1 and FRK2 were rarely changed, while acetylation of BFRUCT1 (A0A1D6H9Z1), UG74B1and GME-1 were slight declined (Fig. 7). But acetylation of these proteins did not change significantly at each time points in Lx9801, except for BFRUCT1s and RT1, which were slightly elevated at 12 h and 24 h (Fig. 7).

#### Antioxidant proteins

Defense mechanisms associated with ROS signalling confer protection to plants following pathogen infection [64]. We observed that acetylation of seven enzymes that scavenge free ROS showed reverse patterns in CML496 and Lx9801 during PP.CN1.0 infection (Fig. 7), including: glutathione S-transferase T1 (GSTT1), glutathione reductase (GR), two isoforms of glutathione synthetases (GSSs) and three peroxidases (PODs) [59]. Acetylation of six oxygen-evolving enhancer proteins (OEEs) used scavenge ROS [65] was decreased at each time point of CML496 and Lx9801 (Fig. 7). Four aquaporins (PIP1–1, PIP2–1, and two isoforms of PIP2–2) involved in pathogen-induced H_2_O_2_ transport [66] were also differentially regulated by Kac (Fig. 6). Analysis of basal metabolic proteins involved in ROS production and antioxidant defence revealed that Kac of these proteins was differentially regulated in both CML496 and Lx9801 (Fig. S10, Tables S3 and S4).

#### Proteostasis proteins

To constantly maintain cellular protein homeostasis of cells in response to external environment stimuli, a dynamic proteostasis network is used to guarantee protein successful synthesis, correct folding and degradation [67]. Dozens of proteins including the ubiquitin-like protein RUB1 and UBL5, the E1 ubiquitin-activating enzyme UBA, the E3 ubiquitin transferases CUL1 and CUL3A, the proteasome activator subunit 4 (PSME4), the proteasome subunit alpha (PSMA), the cathepsin B-like cysteine proteinase 3 (CP3), the serine protease SPPA, the senescence-specific cysteine protease SAG39, and two disulfide-isomerases (PDIs) that catalyze the formation of disulfide bonds in proteins to ensure the correct conformation of the protein [68], were found to be acetylated, and the acetylation of these proteins showed almost reverse patterns in CML496 and Lx9801 under PP.CN1.0 infection (Fig. 7, S10).

Further analysis of the quantitative acetylome data revealed that translation relevant proteins were also differentially acetylated in CML496 and Lx9801 infected with PP.CN1.0, and their acetylation showed a reverse pattern (Fig. S10). For example, EIF5A, EIF3F, EIF5, EIF3H, EIF3D and two isoforms of EIF4G1 in translational initiation complex; TEF4, EEF2, TEF1, and two isoforms of EFG in translational elongation complex; and lysine-tRNA ligase, histidine-tRNA ligase, valy-tRNA ligase, asparagine-tRNA ligase and methionyl-tRNA ligase in translation machinery (Fig. S10). Moreover, transcriptional reprogramming associated proteins, including ADA2b, TFIID5, CAMTA2, HEC1, GLK1, bHLH96, PAB2, MBF1-TF and MOM1, were also differentially acetylated in CML496 and Lx9801 (Fig. S10).

## Discussion

### Features of lysine acetylome in plant immune response

Protein acetylation is a ubiquitous PTM, found in both eukaryotes and prokaryotes, and is involved in various key cellular processes [3, 69]. Expression, biosynthesis and secretion of pathogenic factors in pathogens are regulated by protein acetylation during the interaction between pathogens and host plants [70]. Walley et al. previously revealed that the activity of plant-encoded enzymes can be modulated to alter acetylation of nonhistone proteins during immune response using HCT-deficient (Tox^−^) and HCT-producing (Tox^+^) strain of C. *carbonum* race 1 [28]. However, the regulation of lysine acetylation in the immune response or defense activity of plants is unclear, especially the pathogenic process from germination to establishment of pathogens. Here, we used LC-MS/MS-based global maize acetylome profiling to identify 7412 Kac sites from 4697 proteins in SCR-resistant and -susceptible maize infected with *P. polysora* for 0 h, 12 h, 24 h, 48 h and 72 h, and quantify 1732 Kac sites of 1006 proteins (Fig. 1C, D and Table S2, S3). This represents a 1 ~ 4-fold increase in maize acetylome relative to previous reports [28, 31], allowing us to analyze proteome-wide features of Kac during plant-pathogen interactions. In addition, we found defense-associated pathways were particularly enriched in pathogen-infected maize, such as the ascorbate metabolism, oxocarboxylic acid metabolism, monobactam biosynthesis, linoleic acid metabolism and endocytosis (Fig. 2E), which have not been identified in previous reports.

Moreover, the ε-amino group of lysine is positively charged at physiological pH and can be used for ionic interactions, for hydrogen bonding, or as a general base in enzyme catalysis [71, 72]. Several recent studies in mammals and bacteria have shown that the stoichiometry of lysine acetylation is low at the proteome level and that some acetylation sites are catalyzed by non-enzymatic mechanisms due to the deprotonation of the ε-amino group at high pH that facilitates its nucleophilic attack on acetyl-CoA [73, 74]. The majority of Kac proteins in mammalian mitochondria are non-enzymatically acetylated at a pH of around 8 in the matrix [75]. This raises the concern that some Kac sites are due to stochastic non-enzymatic acetylation. However, several pieces of evidence support our determination that a large portion of the Kac sites identified here are catalyzed by the enzyme. First, subcellular localization analysis indicated that the most localized Kac proteins are in the nucleus (Fig. 2B), consistent with the location of most KATs and KDACs. Second, the next two highest amounts of Kac proteins localization are in chloroplast and cytosol (Fig. 2B), both of which have a pH around 7.2 in Arabidopsis [76]. Third, the number of mitochondrial Kac proteins at pH 8.0 is similar to that of the endomembrane system and extracellular space at pH 5.2 to 7.1 (Fig. 2B) [76]. In addition, in principle negatively charged residues could help deprotonate lysine and facilitate acetylation reactions [71, 72]. But motif analyses revealed a clear preference of amino acids surrounding Kac sites in different organelles (Fig. 2C and Fig. S2), which may reflect the preference of KAT and KDAC enzymes. The − 3 position at the upstream of the Kac site and + 1 position next to the Kac site may be most important for acetylation. All non-polar hydrophobic amino acids including Phe (F) and Trp (W), as well as the polar neutral amino acid Tyr (Y), a target amino acid for phosphorylation, are significantly overrepresented at − 3 position in all organelles (Fig. 2C, Fig. S2). This position has previously been showed to be a part of the acetylation of mitochondrial proteins [77]. In contrast, amino acids at + 1 position prefer to polar neutral residues, including Asn (N), Thr (T), Ser (S), Tyr (Y), Pro (P) and Gly (G) (Fig. 2C, Fig. S2). In rat and Arabidopsis, however, Kac preferentially occurs in Lys-rich regions with negatively charged amino acids surrounding the Kac sites [30, 77]. These differences in these motif patterns suggest that the -F/W/Y-X-X-K (ac)-N/S/T/P/Y/G- motif may tend to regulate immunoregulatory-related proteins in pathogenic infection.

In addition to the altered levels of histone acetylation that occur during the immune response, pathogen infections have been shown to induce non-histone protein acetylation and alter host immunity. GO and KEGG analysis revealed that the identified Kac proteins represent a wide range of functional categories (Fig. 2D, E and Fig. S3), suggesting that Kac can regulate diverse biological processes in the pathogen response. Analysis of biological process of acetylome in SCR-resistant and -susceptible maize, the results show that Kac preferentially targets proteins involved in energy process, metabolic processes, ion transport and signaling transduction processes (Fig. 3B). Moreover, we observed that a specific enrichment of SCR-resistant specific Kac proteins in CML496 in the endocytosis and plant-pathogen interaction pathways (Fig. 3C), and that many proteins responsible for major commercial traits were also acetylated, such as starch, sucrose and amino acid biosynthesis (Fig. 3C). These results suggest that lysine acetylation has a regulatory role on immune response and growth during pathogen infection. Quantitative analysis further revealed that acetylation of histone and non-histone were dynamically regulated during infection, and that acetylation of proteins presented a reverse pattern (Figs. 4 and 5 and Table S3).

Fungal infection requires successful germination, penetration, establishment of urediniospores. We found that proteins with down-regulated acetylation were specifically enriched in the amino acid biosynthesis, spliceosome, endocytosis and biotin metabolism in SCR-susceptible maize infected with PP.CN1.0 for 12 h compared to SCR-resistant maize (Fig. 4D). As infection proceeds, acetylation of proteins involved in these pathways was up-regulated at 48 h, along with acetylation of amino acid metabolism proteins associated with defense (Fig. 4C). In contrast, acetylation of proteins involved in these pathways was unchanged in SCR-resistant maize (Fig. 4C, D). Acetylation of proteins involved in plant-pathogen interaction at 24 h and 48 h of PP.CN1.0 infection, proteins involved in endoplasmic reticulum protein processing at 24 h of infection, proteins involved in ubiquinone biosynthesis at 48 h of infection, and proteins involved in biosynthesis of cofactors at 48 h and 72 h of infection, were specifically up-regulated in SCR-resistant maize (Fig. 4C). At the late stages of PP.CN1.0 colonization (at 72 h) in SCR-susceptible maize, up-regulated Kac proteins were specifically enriched in photosynthesis and oxidative phosphorylation pathways, and down-regulated Kac proteins were enriched in amino acid metabolism pathways, whereas protein acetylation of these pathways showed reverse patterns in SCR-resistant maize (Fig. 4C, D). These findings, together with previously published reports that fungal pathogen infection results in biosynthesis of carbohydrates, redistribution of amino acids and metabolites [78], suggest that lysine acetylation may dynamically regulate plant metabolism and immune defense against fungal pathogens.

### Protein acetylation plays a critical role in the regulation of plant immune response during pathogenesis

**Fig. 8:**
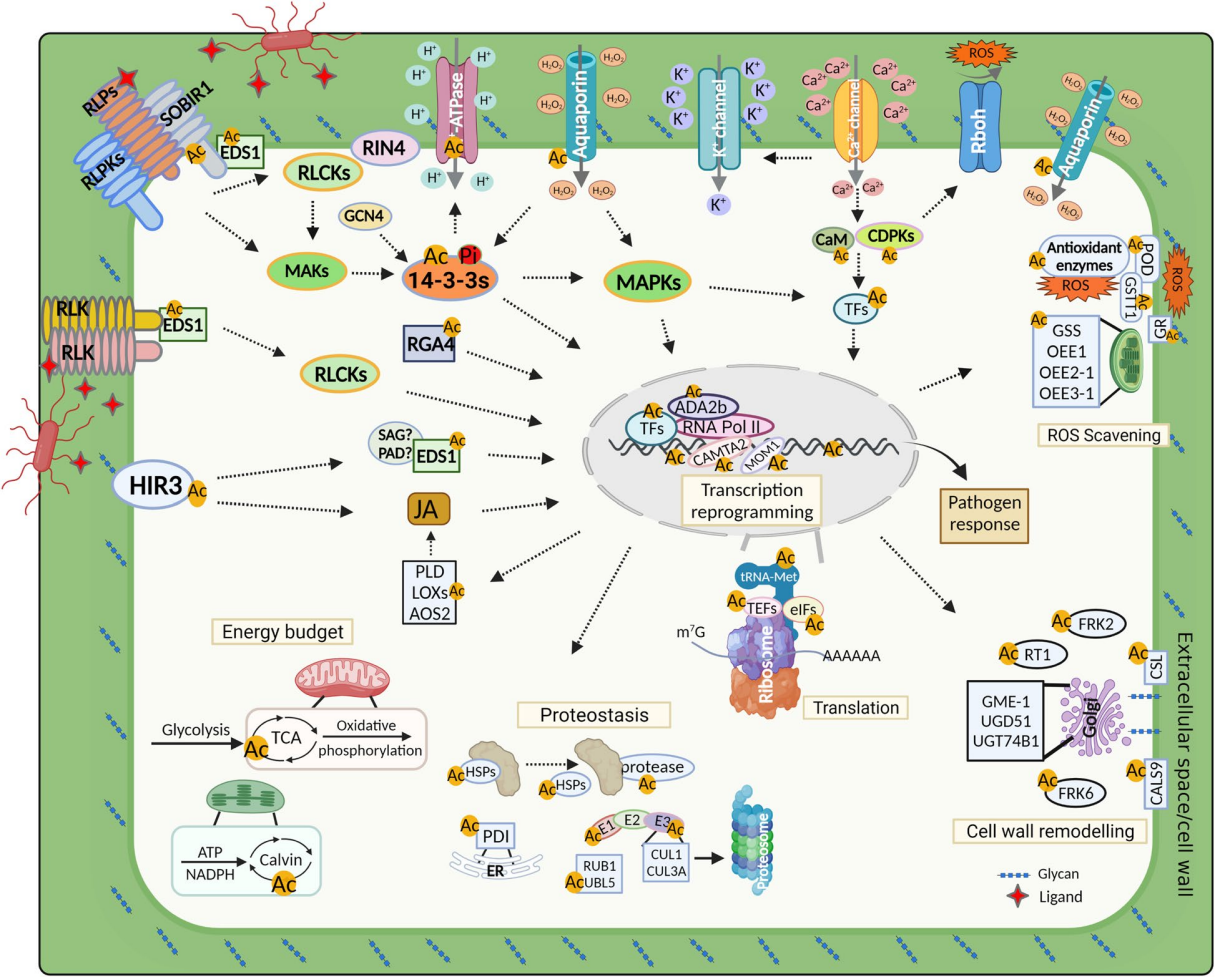
A proposed model for defense against *P. polysora* attack in maize (created with BioRender.com). Abbreviations: RLPs, receptor-like proteins; RLPKs, receptor-like protein kinases; RLK, receptor-like kinase; RLCKs, receptor-like cytoplasmic kinases; SOBIR1, LRR-like Ser/Thr/Tyr-protein kinase SOBIR1; RIN4, RPM1-interacting protein 4; EDS1, Enhanced disease susceptibility 1; GCN4, GENERAL CONTROL NONREPRESSIBLE 4; 14–3-3 s, 14–3-3 proteins; RGA4, R-GENE ANALOG 4; HIR3, Hypersensitive induced reaction 3; CaM, calmodulin; CDPKs, calcium dependent protein kinases; TFs, transcription factors; JA, Jasmonic acid; POD, peroxidase; GSTT1, Glutathione S-transferase T1; GSS, glutathione synthetase; OEE1, oxygen-evolving enhancer protein 1; OEE2–1, oxygen-evolving enhancer protein 2–1; OEE3–1, oxygen-evolving enhancer protein 3–1; CALS9, callose synthase 9; CSL, cellulose synthase-like family protein; FRK2, fructokinase-2; FRK6, fructokinase-6; RT1, UDP-rhamnose,rhamnosyltransferase 1; GME-1, GDP-mannose 3,5-epimerase 1; UGT74B1, UDP-glycosyltransferase 74B1; UGD51, UDP-glucose 6-dehydrogenase; eIFs, eukaryotic initiation factors; TEFs, transcription elongation factors; HSPs, heat shock proteins; PDI, protein disulfide-isomerase; UBL5, ubiquitin-like protein 5; RUB1, Ubiquitin-NEDD8-like protein RUB1; E1, ubiquitin-activating enzyme; E2, ubiquitin conjugating enzyme; E3, ubiquitin transferase; CUL1, cullin-1; CUL3A, cullin-3A; TAC, tricarboxylic acid cycle; Calvin, calvin cycle; SAG, senescence-associated carboxylesterase; PAD, phytoalexin

With the role in neutralizing the positive charge of lysine and introducing steric hindrance, Kac can affect various functions of the targets, including regulating enzymatic activity [79], altering subcellular localization [80], protein stability [81], cross-talk with other PTMs [30] and protein-protein/DNA interactions [82]. Bioinformatics analysis revealed that this fungal induced-specific acetylome data set provides evidence that site-specific Kac proteins may be involved in multiple immune category functions with which the modification has not previously been associated (Figs. 6, 7 and 8). For example the cell surface-localized receptors, including LRR-like protein kinase SOBIR1, hypersensitive induced reaction protein HIR3, NB-LRR disease resistance protein RGA4 and TNL-mediated resistance response required membrane-anchoring EDS1, can detect extracellular signaling molecules result in intracellular responses [18, 83]. SOBIR1 interacts with LRR-RLP Cf-4 or RPL23 and BAK1 form a tripartite complex to trigger activation of RLCKs induce immune response [40, 84], and SOBIR1 also links RLP23 with EDS1, PAD4 and ADR1 to form a supramolecular complex that induces downstream immune signaling transduction [85]. EDS1 shuttles between the cytoplasm and nucleus via receptor-mediated nuclear transport [86], and interacts with PAD4 or SAG101 modules to response to various biotic stresses [41] and with RPS4 and other components to regulate transcriptional reprogramming [87]. Moreover, HIR3, which is involved in cell death, also triggers immune response via EDS1-driven basal resistance and SA-dependent pathways [42]. We also found that PLD, allene oxide synthase 2 and six lipoxygenases involved in SA synthesis were differentially regulated by Kac upon *P. polysora* infection (Fig. 6). Previous study demonstrated that mutation of Lys56 of 14–3-3λ/GRF6 to Gln (Q, acetyllysine mimic residue) inhibits it interaction with phosphorylated AHA2 to regulate the alkaline response [30]. In addition, 14–3-3 s can also modulate stomatal aperture to control pathogens invasion by directly binding to RIN4 and PM H^+^-ATPase AHA2 via GCN4 [50, 51]. We found that acetylation of 12 Kac sites in 14–3-3 s were also differentially regulated in CML496 and Lx9801, with Lys56 being elevated in CML496 and decreased in Lx9801 after infection with PP.CN1.0 (Table S4). Moreover, we found that two lysine residues in AHA2 were also acetylated and their levels were slightly reduced in CML496 and increased in Lx9801 at 48 h and 72 h of *P. polysora* infection (Fig. 6, Table S4). On the other hand, our data also showed that acetylation of quantified calcium-mediated signaling proteins were almost all up-regulated in both CML496 and Lx9801 infected with *P. polysora* (Fig. 7). This suggests that acetylation may regulate activity of immune relevant membrane proteins and intracellular proteins in response to pathogen invasion (Fig. 8).

Activation of defense upon recognition of exogenous pathogen-derived PAMPs or endogenous danger signals (i.e., DAMPs) is a key function of the plant innate immunity [83]. Plant cell walls are a source of oligosaccharide fragments with signaling functions for development and immunity. For example, the breakage products of the homogalacturonan (HG) of cell-wall, oligogalacturonides (OGs), are released by pectin-degrading enzymes of microbes and are perceived as DAMPs for defense against microbes and local signal for repair mechanical injuries [88]. Evidence supports the existence of a plant cell-wall integrity maintenance system [89]. We found that proteins involved in cell-wall remodeling, including fructokinases FRK6 and FRK2, cellulose synthases CALS9 and CSL, cell wall isozyme BRFUCT1, and enzymes for the interconversion of nucleotide sugars required for cell-wall biosynthesis, including GME1, RT1, UGD51 and UGT74B1, were all regulated by Kac after *P. polysora* infection (Fig. 6). Among them, fructokinase has been implicated as an enzyme required for carbon partitioning from fructose to cell-wall polysaccharides [61]. Previous studies also showed that pathogen secreted effectors acetylate tubulin, disrupting the microtubule network and thereby inhibiting cell wall-mediated defence responses [7, 90]. This suggests that Kac is involved not only in pathogen-induced cell-wall destruction but also in the regulation of cell-wall biosynthesis to control cell-wall integrity during pathogen infection (Fig. 8).

Production of ROS is critical for successful activation of immune response against pathogen infection, and plays important roles in both PTI and ETI [91]. However, the excessive accumulation of ROS can also damage cell membranes and proteins. To alleviate the ROS damage, plants have evolved a series of antioxidant enzymes [92]. We found that acetylation of three free ROS-scavenging enzymes PODs decreased in CML496 and increased in Lx9801, the acetylation of two different organelle localized thioredoxin-dependent peroxiredoxins presented reverse acetylation patterns in CML496 and Lx9801 (Fig. 7), and the acetylation of six additional ROS-scavenging protein OEEs decreased in both CML496 and Lx9801 (Fig. 7). In addition, we found that four aquaporins involved in pathogen-induced H_2_O_2_ transport [66] were also differentially regulated by Kac (Fig. 6) These findings suggest that Kac may response to pathogenic attack by regulating the activity of ROS scavenging-related proteins (Fig. 8).

Activation of plant immune response induces the accumulation of large amounts of immune-associated proteins. Unfortunately, the accumulation of unfolded/misfolded proteins is toxic to cells [93]. Enriched GO terms show that fungal-induced Kac proteins are concentrated in macromolecular complexes such as ribonucleoprotein complex, protein-DNA complex, DNA packing complex and ribosome, and are involved in the regulation of translation, unfolding protein, protein heterodimerization and nucleoside binding activity (Fig. 2D). Quantitative analysis further revealed potential functions of Kac in redox reaction, proteostasis, translation and transcriptional reprogramming (Fig. 7, Fig. S10). This suggests that Kac may control reprogramming of transcription, protein synthesis, folding and degradation to combat pathogenic attack (Fig. 8).

## Conclusion

Here, we identified a total of 7412 Kac site in 4697 proteins from maize, together with previously published acetylome dataset, bring the number of Kac sites to the same magnitude as the number of phosphorylation sites. In addition, our acetylome dataset also highlights various cellular events involving lysine acetylation proteins, which stresses the important of mapping protein modifications in the pathogenesis of pathogenic infections. Among these findings, we observed that acetylation of cellular events associated proteins showed reverse patterns in SCR-resistant and -susceptible maize. Not only do we find that specific proteins and sites associated with immunity are differentially acetylated in SCR-resistant and -susceptible maize, we also show that the distribution of Kac proteins among subcellular compartments is more likely to be an enzymatic rather than a non-enzymatic mechanism. Based on the previous discussion, a hypothetical model is proposed to illustrate cellular events potentially involvement of Kac associated with defense against fungal attack in maize (Fig. 8).

## Supplementary Information

**Additional file 1: Table S1–1.** Information on proteins of maize infected with P. polysora quantified based on a DIA-quantitative proteomics approach. **Table S1–2.** Information on peptides of maize infected with P. polysora quantified by Spectronaut.

**Additional file 2: Table S2–1.** Information on acetylated peptides of maize infected by P. polysora infection identified by pFind. **Table S2–2.** Information on acetylated sites in maize infected by P. polysora identified by pFind and Walley. et.al. and Yan. et.al. **Table S2–3.** Information on acetylated proteins in maize infected by P. polysora identified by pFind and Walley. et.al. and Yan. et.al.

**Additional file 3: Table S3–1.** Quantitative information of acetylated PMSs of maize with P. polysora infection quantified by Protein Discovery. **Table S3–2.** Quantitative information of acetylated peptides of maize with P. polysora infection. **Table S3–3.** Quantitative information of acetylated sites of maize with P. polysora infection. **Table S3–4.** Quantitative information of acetylated proteins of maize with P. polysora infection.

**Additional file 4: Table S4.** Defense-associated Kac site involved in invasion of P. polysora.

**Additional file 5: Figure S1.** GO (gene ontology) and KEGG enrichment analysis of shared Kac proteins identified by Walley et al. and this study. **Figure S2.** Sequence logo of identified Kac sites with all proteins as background population in nucleus (**A**), cytoplasm (**B**), chloroplast (**C**), mitochondrion (**D**), extracellular space (**E**), endomembrane system (**F**), plasma membrane (**G**) and organelle membrane (**H**) (generated using pLogo). **Figure S3.** GO (gene ontology) enrichment analysis of all identified Kac proteins. **Figure S4.** Distribution of Kac proteins (**A**) and sites (**B**) in SCR-resistant and susceptible maize infected with *P. polysora* for 0-h, 12-h, 24-h, 48-h and 72-h. **Figure S5.** Molecular function enrichment analysis of all identified common and specific Kac proteins in CML496 and Lx9801. **Figure S6.** Cellular component enrichment analysis of all identified common and specific Kac proteins in CML496 and Lx9801. **Figure S7.** Fold changes in proteins and Kac sites of CML496 treated with *P. polysora* for 12 h (**A**), 24 h (**B**), 48 h (**C**) and 72 h (**D**) compared to 0 h. Fold changes in proteins and Kac sites of Lx9801 treated with *P. polysora* for 12 h (**E**), 24 h (**F**), 48 h (**G**) and 72 h (**H**) compared to 0 h. **Figure S8.** Biological process enrichment analyses of up-regulated (**A**) and down-regulated (**B**) DKPs in CML496 and Lx9801 with *P. polysora* infection for 12 h, 24 h, 48 h and 72 h compared to 0 h. **Figure S9.** Sequence alignment of histone H3 (**A**) and H4 (**B**)**.** Asterisks (*) indicate conserved amino acid sites, dots (·) indicate relatively conserved amino acids, colons (:) indicate slightly conserved amino acid. **Figure S10.** Heat map of mainly differential acetylated proteins (DAPs) involved in redox reaction, kinase activity, transcription and translation found in SCR-resistant and susceptible maize infected with *P. polysora.*

**Additional file 6.**

## Data Availability

The data sets supporting the results of this article are included within the article and its additional files. Raw data files can be download from the UCSD MassIVE mass spectrometry data repository (https://massive.ucsd.edu) using the identifier MSV000088717.

## Abbreviations

14–3-3 s: 14–3-3 proteins
CALS9: Callose synthase 9
Calvin: Calvin cycle
CaM: Calmodulin
CDPKs: Calcium dependent protein kinases
CSL: Cellulose synthase-like family protein
CUL1: Cullin-1
CUL3A: Cullin-3A
E1: Ubiquitin-activating enzyme
E2: Ubiquitin conjugating enzyme
E3: Ubiquitin transferase
EDS1: Enhanced disease susceptibility 1
eIFs: eukaryotic initiation factors
FRK2: Fructokinase-2
FRK6: Fructokinase-6
GCN4: GENERAL CONTROL NONREPRESSIBLE 4
GME-1: GDP-mannose 3,5-epimerase 1
GSS: Glutathione synthetase
GSTT1: Glutathione S-transferase T1
HIR3: Hypersensitive induced reaction 3
HSPs: Heat shock proteins
JA: Jasmonic acid
OEE: Oxygen-evolving enhancer
PAD: Phytoalexin
PDI: Protein disulfide-isomerase
POD: Peroxidase
RGA4: R-GENE ANALOG 4
RIN4: RPM1-interacting protein 4
RLCKs: Receptor-like cytoplasmic kinases
RLK: Receptor-like kinase
RLPKs: Receptor-like protein kinases
RT1: UDP-rhamnose,rhamnosyltransferase 1
RUB1: Ubiquitin-NEDD8-like protein RUB1
SAG: Senescence-associated carboxylesterase
SOBIR1: LRR-like Ser/Thr/Tyr-protein kinase SOBIR1
TAC: Tricarboxylic acid cycle
TEFs: Transcription elongation factors
TFs: Transcription factors
UBL5: Ubiquitin-like protein 5
UGD51: UDP-glucose 6-dehydrogenase
UGT74B1: UDP-glycosyltransferase 74B1
RLPs: Receptor-like proteins

## References

[1] Phillips DM (1963). The presence of acetyl groups of histones. Biochem J.

[2] Diallo I, Seve M, Cunin V, Minassian F, Poisson JF, Michelland S, Bourgoin-Voillard S (2019). Current trends in protein acetylation analysis. Expert Rev Proteomics.

[3] Xia L, Kong X, Song H, Han Q, Zhang S (2022). Advances in proteome-wide analysis of plant lysine acetylation. Plant Com.

[4] Narita T, Weinert BT, Choudhary C (2019). Functions and mechanisms of non-histone protein acetylation. Nat Rev Mol Cell Biol.

[5] Xing S, Poirier Y (2012). The protein acetylome and the regulation of metabolism. Trends Plant Sci.

[6] Wang J, Liu C, Chen Y, Zhao Y, Ma Z (2021). Protein acetylation and deacetylation in plant-pathogen interactions. Environ Microbiol.

[7] Lee AH, Hurley B, Felsensteiner C, Yea C, Ckurshumova W, Bartetzko V, Wang PW, Quach V, Lewis JD, Liu YC (2012). A bacterial acetyltransferase destroys plant microtubule networks and blocks secretion. PLoS Pathog.

[8] Lee J, Manning AJ, Wolfgeher D, Jelenska J, Cavanaugh KA, Xu H, Fernandez SM, Michelmore RW, Kron SJ, Greenberg JT (2015). Acetylation of an NB-LRR Plant immune-effector complex suppresses immunity. Cell Rep.

[9] Brosch G, Ransom R, Lechner T, Walton JD, Loidl P (1995). Inhibition of maize histone deacetylases by HC toxin, the host-selective toxin of Cochliobolus carbonum. Plant Cell.

[10] Xu Q, Wang J, Zhao J, Xu J, Sun S, Zhang H, Wu J, Tang C, Kang Z, Wang X (2020). A polysaccharide deacetylase from Puccinia striiformis f. sp. tritici is an important pathogenicity gene that suppresses plant immunity. Plant Biotechnol J.

[11] Sarris PF, Duxbury Z, Huh SU, Ma Y, Segonzac C, Sklenar J, Derbyshire P, Cevik V, Rallapalli G, Saucet SB (2015). A plant immune receptor detects pathogen effectors that target WRKY transcription factors. Cell.

[12] Le Roux C, Huet G, Jauneau A, Camborde L, Tremousaygue D, Kraut A, Zhou B, Levaillant M, Adachi H, Yoshioka H (2015). A receptor pair with an integrated decoy converts pathogen disabling of transcription factors to immunity. Cell.

[13] Tasset C, Bernoux M, Jauneau A, Pouzet C, Briere C, Kieffer-Jacquinod S, Rivas S, Marco Y, Deslandes L (2010). Autoacetylation of the Ralstonia solanacearum effector PopP2 targets a lysine residue essential for RRS1-R-mediated immunity in Arabidopsis. PLoS Pathog.

[14] Huh SU. PopP2 interacts with PAD4 in an acetyltransferase activity-dependent manner and affects plant immunity. Plant Signal Behav. 2021;6(12):e2017631(1–5). 10.1080/15592324.2021.2017631 PMC9208800 34978271

[15] Lin W, Ma X, Shan L, He P (2013). Big roles of small kinases: the complex functions of receptor-like cytoplasmic kinases in plant immunity and development. J Integr Plant Biol.

[16] Rui Y, Dinneny JR (2020). A wall with integrity: surveillance and maintenance of the plant cell wall under stress. New Phytol.

[17] Bacete L, Melida H, Miedes E, Molina A (2018). Plant cell wall-mediated immunity: cell wall changes trigger disease resistance responses. Plant J.

[18] Ngou BPM, Jones JDG, Ding P (2022). Plant immune networks. Trends Plant Sci.

[19] Zhou JM, Zhang Y (2020). Plant immunity: danger perception and signaling. Cell.

[20] Debnath S, Chhetri S, Biswas S (2019). Southern rust disease of corn – a review. Int J Curr Microbiol App Sci.

[21] Sun Q, Li L, Guo F, Zhang K, Dong J, Luo Y, Ma Z (2021). Southern corn rust caused by Puccinia polysora Underw: a review. Phyt Research.

[22] Keller B, Feuillet C, Messmer M, Slusarenko AJ, RSS F, van Loon LC (2000). Genetics of disease resistance. Mech Resis Plant Dis.

[23] Wang Q, Zhang Y, Yang C, Xiong H, Lin Y, Yao J, Li H, Xie L, Zhao W, Yao Y (2010). Acetylation of metabolic enzymes coordinates carbon source utilization and metabolic flux. Science.

[24] Zhao S, Xu W, Jiang W, Yu W, Lin Y, Zhang T, Yao J, Zhou L, Zeng Y, Li H (2010). Regulation of cellular metabolism by protein lysine acetylation. Science.

[25] Christensen SA, Santana EA, Alborn HT, Block AK, Chamberlain CA (2021). Metabolomics by UHPLC-HRMS reveals the impact of heat stress on pathogen-elicited immunity in maize. Metabolomics.

[26] Wan L, Essuman K, Anderson RG, Sasaki Y, Monteiro F, Chung EH, Osborne Nishimura E, DiAntonio A, Milbrandt J, Dangl JL (2019). TIR domains of plant immune receptors are NAD^+^-cleaving enzymes that promote cell death. Science.

[27] Horsefield S, Burdett H, Zhang X, Manik MK, Shi Y, Chen J, Qi T, Gilley J, Lai JS, Rank MX (2019). NAD^+^ cleavage activity by animal and plant TIR domains in cell death pathways. Science.

[28] Walley JW, Shen Z, McReynolds MR, Schmelz EA, Briggs SP (2018). Fungal-induced protein hyperacetylation in maize identified by acetylome profiling. Proc Natl Acad Sci U S A.

[29] Deng C, Leonard A, Cahill J, Lv M, Li Y, Thatcher S, et al. The RppC-AvrRppC NLR-effector Interaction Mediates the resistance to Southern Corn Rust in Maize. Mol Plant. 2022;15(5):904–12. 10.1016/j.molp.2022.01.007 35032688

[30] Guo J, Chai X, Mei Y, Du J, Du H, Shi H, Zhu J-K, Zhang H (2022). Acetylproteomics analyses reveal critical features of lysine-ε-acetylation in Arabidopsis and a role of 14-3-3 protein acetylation in alkaline response. Stress Biol.

[31] Yan Z, Shen Z, Gao ZF, Chao Q, Qian CR, Zheng H, Wang BC (2020). A comprehensive analysis of the lysine acetylome reveals diverse functions of acetylated proteins during de-etiolation in Zea mays. J Plant Physiol.

[32] Isaacson T, Damasceno CM, Saravanan RS, He Y, Catala C, Saladie M, Rose JK (2006). Sample extraction techniques for enhanced proteomic analysis of plant tissues. Nat Protoc.

[33] O'Shea JP, Chou MF, Quader SA, Ryan JK, Church GM, Schwartz D (2013). pLogo: a probabilistic approach to visualizing sequence motifs. Nat Methods.

[34] Kanehisa M, Furumichi M, Tanabe M, Sato Y, Morishima K (2017). KEGG: new perspectives on genomes, pathways, diseases and drugs. Nucleic Acids Res.

[35] Zhao YG, Zhang H (2020). Phase separation in membrane biology: the interplay between membrane-bound organelles and Membraneless condensates. Dev Cell.

[36] Savojardo C, Martelli PL, Fariselli P, Profiti G, Casadio R (2018). BUSCA: an integrative web server to predict subcellular localization of proteins. Nucleic Acids Res.

[37] Choudhary C, Weinert BT, Nishida Y, Verdin E, Mann M (2014). The growing landscape of lysine acetylation links metabolism and cell signalling. Nat Rev Mol Cell Biol.

[38] Jiang K, Duan C, Wu X, Yang Z, Wang X (2015). Histology and ultrastructure of maize lines infected by Puccinia polysora. J Plant Protect.

[39] Choi S, Prokchorchik M, Lee H, Gupta R, Lee Y, Chung EH, Cho B, Kim MS, Kim ST, Sohn KH (2021). Direct acetylation of a conserved threonine of RIN4 by the bacterial effector HopZ5 or AvrBsT activates RPM1-dependent immunity in Arabidopsis. Mol Plant.

[40] Albert I, Bohm H, Albert M, Feiler CE, Imkampe J, Wallmeroth N, Brancato C, Raaymakers TM, Oome S, Zhang H (2015). An RLP23-SOBIR1-BAK1 complex mediates NLP-triggered immunity. Nat Plants.

[41] Lapin D, Bhandari DD, Parker JE (2020). Origins and immunity networking functions of EDS1 family proteins. Annu Rev Phytopathol.

[42] Li S, Zhao J, Zhai Y, Yuan Q, Zhang H, Wu X, Lu Y, Peng J, Sun Z, Lin L (2019). The hypersensitive induced reaction 3 (HIR3) gene contributes to plant basal resistance via an EDS1 and salicylic acid-dependent pathway. Plant J.

[43] Hutin M, Cesari S, Chalvon V, Michel C, Tran TT, Boch J, Koebnik R, Szurek B, Kroj T (2016). Ectopic activation of the rice NLR heteropair RGA4/RGA5 confers resistance to bacterial blight and bacterial leaf streak diseases. Plant J.

[44] Cesari S, Kanzaki H, Fujiwara T, Bernoux M, Chalvon V, Kawano Y, Shimamoto K, Dodds P, Terauchi R, Kroj T (2014). The NB-LRR proteins RGA4 and RGA5 interact functionally and physically to confer disease resistance. EMBO J.

[45] Cesari S, Thilliez G, Ribot C, Chalvon V, Michel C, Jauneau A, Rivas S, Alaux L, Kanzaki H, Okuyama Y (2013). The rice resistance protein pair RGA4/RGA5 recognizes the Magnaporthe oryzae effectors AVR-pia and AVR1-CO39 by direct binding. Plant Cell.

[46] Tang C, Wang X, Duan X, Wang X, Huang L, Kang Z (2013). Functions of the lethal leaf-spot 1 gene in wheat cell death and disease tolerance to Puccinia striiformis. J Exp Bot.

[47] Berka M, Kopecká R, Berková V, Brzobohatý B, Černý M (2022). Regulation of heat shock proteins 70 and their role in plant immunity. J Exp Bot.

[48] Campa A, Ferreira JJ (2017). Gene coding for an elongation factor is involved in resistance against powdery mildew in common bean. Theor Appl Genet.

[49] Li DQ, Nair SS, Kumar R (2013). The MORC family: new epigenetic regulators of transcription and DNA damage response. Epigenetics.

[50] Kaundal A, Ramu VS, Oh S, Lee S, Pant B, Lee HK, Rojas CM, Senthil-Kumar M, Mysore KS (2017). GENERAL CONTROL NONREPRESSIBLE4 degrades 14-3-3 and the RIN4 complex to regulate stomatal aperture with implications on nonhost disease resistance and drought tolerance. Plant Cell.

[51] Elmore JM, Coaker G (2011). The role of the plasma membrane H+-ATPase in plant-microbe interactions. Mol Plant.

[52] Shikano I, McCarthy E, Hayes-Plazolles N, Slavicek JM, Hoover K (2018). Jasmonic acid-induced plant defenses delay caterpillar developmental resistance to a baculovirus: slow-growth, high-mortality hypothesis in plant-insect-pathogen interactions. J Invertebr Pathol.

[53] Li J, Wang X (2019). Phospholipase D and phosphatidic acid in plant immunity. Plant Sci.

[54] Pajerowska-Mukhtar KM, Mukhtar MS, Guex N, Halim VA, Rosahl S, Somssich IE, Gebhardt C (2008). Natural variation of potato allene oxide synthase 2 causes differential levels of jasmonates and pathogen resistance in Arabidopsis. Planta.

[55] Shigenaga AM, Argueso CT (2016). No hormone to rule them all: interactions of plant hormones during the responses of plants to pathogens. Semin Cell Dev Biol.

[56] Pollmann S, Springer A, Rustgi S, von Wettstein D, Kang C, Reinbothe C, Reinbothe S (2019). Substrate channeling in oxylipin biosynthesis through a protein complex in the plastid envelope of Arabidopsis thaliana. J Exp Bot.

[57] Liao Z, Wang L, Li C, Cao M, Wang J, Yao Z, Zhou S, Zhou G, Zhang D, Lou Y (2022). The lipoxygenase gene OsRCI-1 is involved in the biosynthesis of herbivore-induced JAs and regulates plant defense and growth in rice. Plant Cell Environ.

[58] Aldon D, Mbengue M, Mazars C, Galaud JP (2018). Calcium signalling in plant biotic interactions. Int J Mol Sci.

[59] Rajput VD, Harish SRK, Verma KK, Sharma L, Quiroz-Figueroa FR, Meena M, Gour VS, Minkina T, Sushkova S (2021). Recent developments in enzymatic antioxidant Defence mechanism in plants with special reference to abiotic stress. Bio.

[60] Jacob P, Kim NH, Wu F, El-Kasmi F, Chi Y, Walton WG, et al. Plant "helper" immune receptors are Ca^2+^-permeable nonselective cation channels. Science. 2021;373(6553):420–5. 10.1126/science.abg7917 PMC8939002 34140391

[61] Roach M, Gerber L, Sandquist D, Gorzsas A, Hedenstrom M, Kumar M, Steinhauser MC, Feil R, Daniel G, Stitt M (2012). Fructokinase is required for carbon partitioning to cellulose in aspen wood. Plant J.

[62] Wang Y, Li X, Fan B, Zhu C, Chen Z. Regulation and function of defense-related Callose deposition in plants. Int J Mol Sci. 2021;22(5):2393(1–15). 10.3390/ijms22052393 PMC7957820 33673633

[63] Douchkov D, Lueck S, Hensel G, Kumlehn J, Rajaraman J, Johrde A, Doblin MS, Beahan CT, Kopischke M, Fuchs R (2016). The barley (Hordeum vulgare) cellulose synthase-like D2 gene (HvCslD2) mediates penetration resistance to host-adapted and nonhost isolates of the powdery mildew fungus. New Phytol.

[64] Baxter A, Mittler R, Suzuki N (2014). ROS as key players in plant stress signalling. J Exp Bot.

[65] Kim EY, Choi YH, Lee JI, Kim IH, Nam TJ (2015). Antioxidant activity of oxygen evolving enhancer protein 1 purified from Capsosiphon fulvescens. J Food Sci.

[66] Zhang M, Shi H, Li N, Wei N, Tian Y, Peng J, Chen X, Zhang L, Zhang M, Dong H (2021). Aquaporin OsPIP2;2 links the H2O2 signal and a membrane-anchored transcription factor to promote plant defense. Plant Physiol.

[67] Orosa B, Üstün S, Calderón Villalobos LIA, Genschik P, Gibbs D, Holdsworth MJ, Isono E, Lois LM, Trujillo M, Sadanandom A (2020). Plant proteostasis – shaping the proteome: a research community aiming to understand molecular mechanisms that control protein abundance. New Phytol.

[68] Wang L, Wang X, Wang CC (2015). Protein disulfide-isomerase, a folding catalyst and a redox-regulated chaperone. Free Radic Biol Med.

[69] Ren J, Sang Y, Lu J, Yao YF (2017). Protein acetylation and its role in bacterial virulence. Trends Microbiol.

[70] Jeon J, Kwon S, Lee YH (2014). Histone acetylation in fungal pathogens of plants. Plant Pathol J.

[71] Luo M (2018). Chemical and biochemical perspectives of protein lysine methylation. Chem Rev.

[72] Smith BC, Denu JM (2009). Chemical mechanisms of histone lysine and arginine modifications. Biochim Biophys Acta.

[73] Tatham MH, Cole C, Scullion P, Wilkie R, Westwood NJ, Stark LA, Hay RT (2017). A proteomic approach to analyze the aspirin-mediated lysine Acetylome. Mol Cell Proteomics.

[74] Weinert BT, Moustafa T, Iesmantavicius V, Zechner R, Choudhary C (2015). Analysis of acetylation stoichiometry suggests that SIRT3 repairs nonenzymatic acetylation lesions. EMBO J.

[75] Wagner GR, Payne RM (2013). Widespread and enzyme-independent Nepsilon-acetylation and Nepsilon-succinylation of proteins in the chemical conditions of the mitochondrial matrix. J Biol Chem.

[76] Shen J, Zeng Y, Zhuang X, Sun L, Yao X, Pimpl P, Jiang L (2013). Organelle pH in the Arabidopsis endomembrane system. Mol Plant.

[77] Lundby A, Lage K, Weinert BT, Bekker-Jensen DB, Secher A, Skovgaard T, Kelstrup CD, Dmytriyev A, Choudhary C, Lundby C (2012). Proteomic analysis of lysine acetylation sites in rat tissues reveals organ specificity and subcellular patterns. Cell Rep.

[78] Aliferis KA, Faubert D, Jabaji S (2014). A metabolic profiling strategy for the dissection of plant defense against fungal pathogens. PLoS One.

[79] Venkat S, Gregory C, Sturges J, Gan Q, Fan C (2017). Studying the lysine acetylation of malate dehydrogenase. J Mol Biol.

[80] Ventura M, Mateo F, Serratosa J, Salaet I, Carujo S, Bachs O, Pujol MJ (2010). Nuclear translocation of glyceraldehyde-3-phosphate dehydrogenase is regulated by acetylation. Int J Biochem Cell Biol.

[81] Jiang W, Wang S, Xiao M, Lin Y, Zhou L, Lei Q, Xiong Y, Guan KL, Zhao S (2011). Acetylation regulates gluconeogenesis by promoting PEPCK1 degradation via recruiting the UBR5 ubiquitin ligase. Mol Cell.

[82] Ren J, Sang Y, Tan Y, Tao J, Ni J, Liu S, Fan X, Zhao W, Lu J, Wu W (2016). Acetylation of lysine 201 inhibits the DNA-binding ability of PhoP to regulate Salmonella virulence. PLoS Pathog.

[83] Ortiz-Morea FA, He P, Shan L, Escocard de Azevedo Manhaes AM (2021). Plant plasma membrane-resident receptors: surveillance for infections and coordination for growth and development. J Integr Plant Biol.

[84] van der Burgh AM, Postma J, Robatzek S, Joosten M (2019). Kinase activity of SOBIR1 and BAK1 is required for immune signalling. Mol Plant Pathol.

[85] Pruitt RN, Locci F, Wanke F, Zhang L, Saile SC, Joe A, Karelina D, Hua C, Frohlich K, Wan WL (2021). The EDS1-PAD4-ADR1 node mediates Arabidopsis pattern-triggered immunity. Nature.

[86] Garcia AV, Blanvillain-Baufume S, Huibers RP, Wiermer M, Li G, Gobbato E, Rietz S, Parker JE (2010). Balanced nuclear and cytoplasmic activities of EDS1 are required for a complete plant innate immune response. PLoS Pathog.

[87] Wirthmueller L, Zhang Y, Jones JD, Parker JE (2007). Nuclear accumulation of the Arabidopsis immune receptor RPS4 is necessary for triggering EDS1-dependent defense. Curr Biol.

[88] De Lorenzo G, Ferrari S, Giovannoni M, Mattei B, Cervone F (2019). Cell wall traits that influence plant development, immunity, and bioconversion. Plant J.

[89] Faria-Blanc N, Mortimer JC, Dupree P (2018). A transcriptomic analysis of Xylan mutants Does not support the existence of a secondary Cell Wall integrity system in Arabidopsis. Front Plant Sci.

[90] Van der Does D, Boutrot F, Engelsdorf T, Rhodes J, McKenna JF, Vernhettes S, Koevoets I, Tintor N, Veerabagu M, Miedes E (2017). The Arabidopsis leucine-rich repeat receptor kinase MIK2/LRR-KISS connects cell wall integrity sensing, root growth and response to abiotic and biotic stresses. PLoS Genet.

[91] Segal LM, Wilson RA (2018). Reactive oxygen species metabolism and plant-fungal interactions. Fungal Genet Biol.

[92] Frendo P, Baldacci-Cresp F, Benyamina SM, Puppo A (2013). Glutathione and plant response to the biotic environment. Free Radic Biol Med.

[93] Verchot J, Pajerowska-Mukhtar KM (2021). UPR signaling at the nexus of plant viral, bacterial, and fungal defenses. Curr Opin Virol.

