# Supplementary material for: A comprehensive dynamic immune acetylproteomics profiling induced by Puccinia polysora in maize
Source: BMC Plant Biol. 2022 Dec 24;22:610. doi: 10.1186/s12870-022-03964-4 (PMC9789614; doi:10.1186/s12870-022-03964-4)
Supplement: Supplementary file 5 — Additional file 5: Figure S1. GO (gene ontology) and KEGG enrichment analysis of shared Kac proteins identified by Walley et al. and this study. Figure S2. Sequence logo of identified Kac sites with all proteins as background population in nucleus (A), cytoplasm (B), chloroplast (C), mitochondrion (D), extracellular space (E), endomembrane system (F), plasma membrane (G) and organelle membrane (H) (generated using pLogo). Figure S3. GO (gene ontology) enrichment analysis of all identified Kac proteins. Figure S4. Distribution of Kac proteins (A) and sites (B) in SCR-resistant and susceptible maize infected with P. polysora for 0-h, 12-h, 24-h, 48-h and 72-h. Figure S5. Molecular function enrichment analysis of all identified common and specific Kac proteins in CML496 and Lx9801. Figure S6. Cellular component enrichment analysis of all identified common and specific Kac proteins in CML496 and Lx9801. Figure S7. Fold changes in proteins and Kac sites of CML496 treated with P. polysora for 12 h (A), 24 h (B), 48 h (C) and 72 h (D) compared to 0 h. Fold changes in proteins and Kac sites of Lx9801 treated with P. polysora for 12 h (E), 24 h (F), 48 h (G) and 72 h (H) compared to 0 h. Figure S8. Biological process enrichment analyses of up-regulated (A) and down-regulated (B) DKPs in CML496 and Lx9801 with P. polysora infection for 12 h, 24 h, 48 h and 72 h compared to 0 h. Figure S9. Sequence alignment of histone H3 (A) and H4 (B). Asterisks (*) indicate conserved amino acid sites, dots (·) indicate relatively conserved amino acids, colons (:) indicate slightly conserved amino acid. Figure S10. Heat map of mainly differential acetylated proteins (DAPs) involved in redox reaction, kinase activity, transcription and translation found in SCR-resistant and susceptible maize infected with P. polysora. [file 12870_2022_3964_MOESM5_ESM.docx]

**Fig. S1** GO (gene ontology) and KEGG enrichment analysis of shared Kac proteins identified by Walley et al. and this study.**
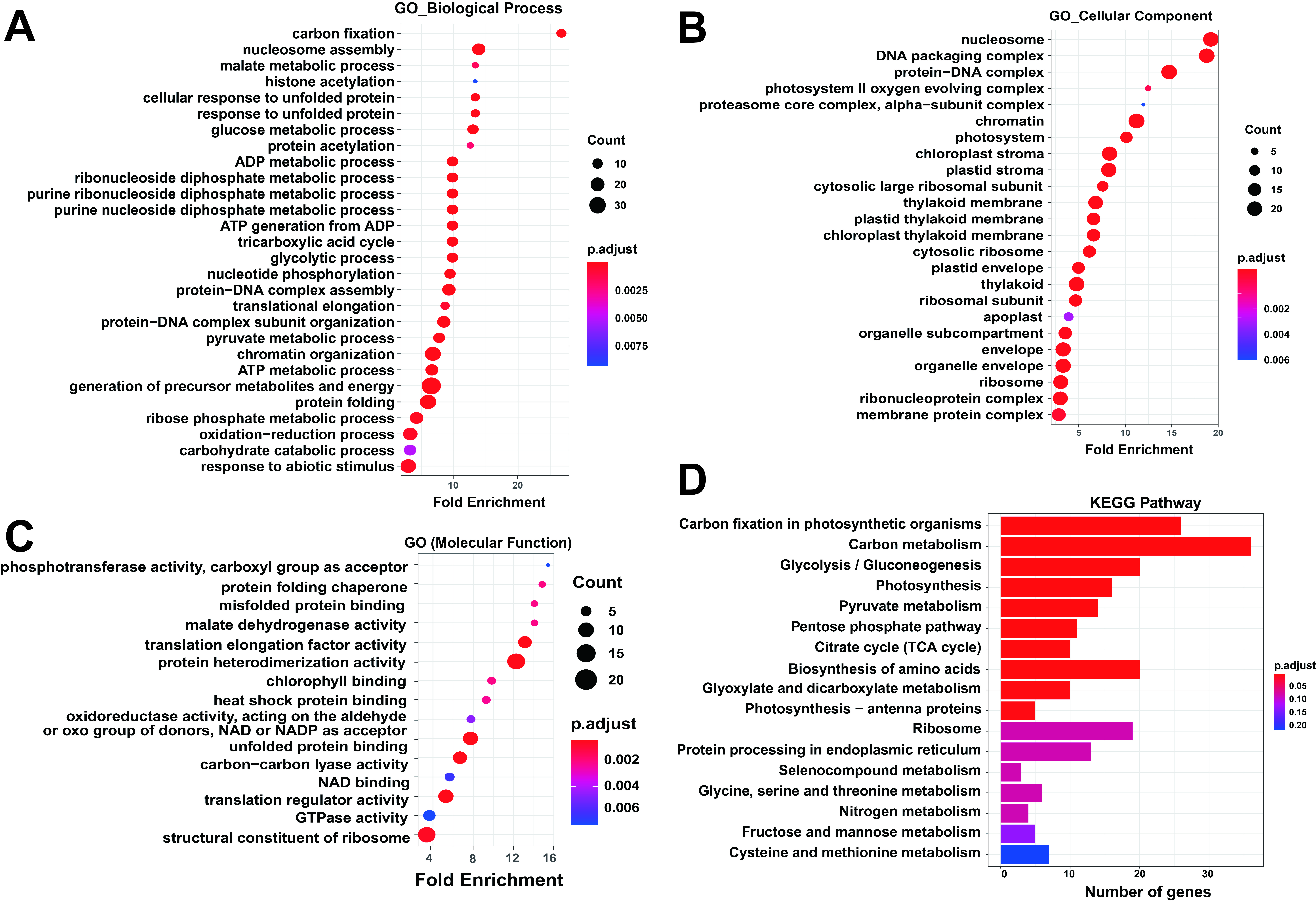
**

**
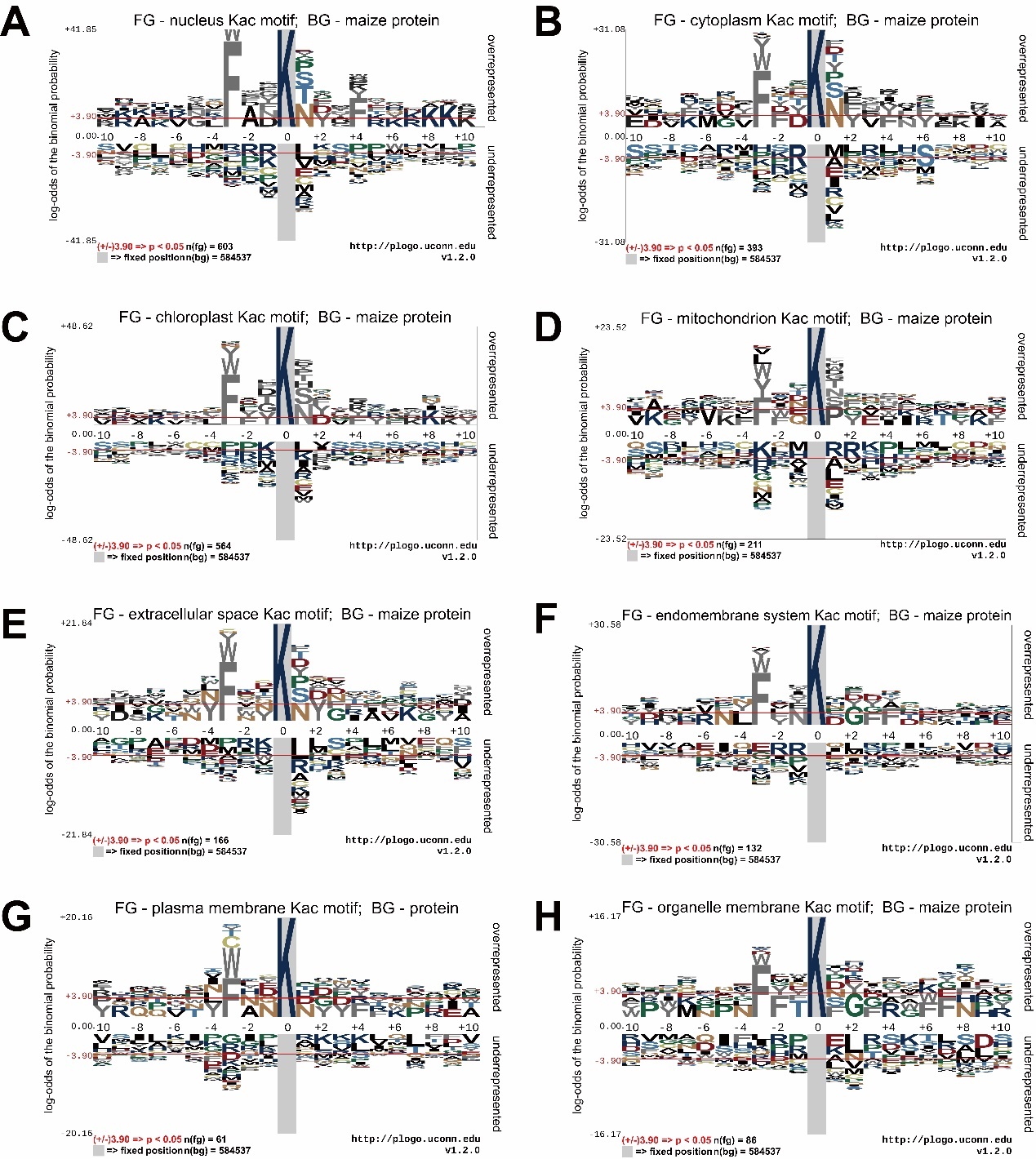
Fig. S2** Sequence logo of identified Kac sites with all proteins as background population in nucleus (**A**), cytoplasm (**B**), chloroplast (**C**), mitochondrion (**D**), extracellular space (**E**), endomembrane system (**F**), plasma membrane (**G**) and organelle membrane (**H**) (generated using pLogo).

**
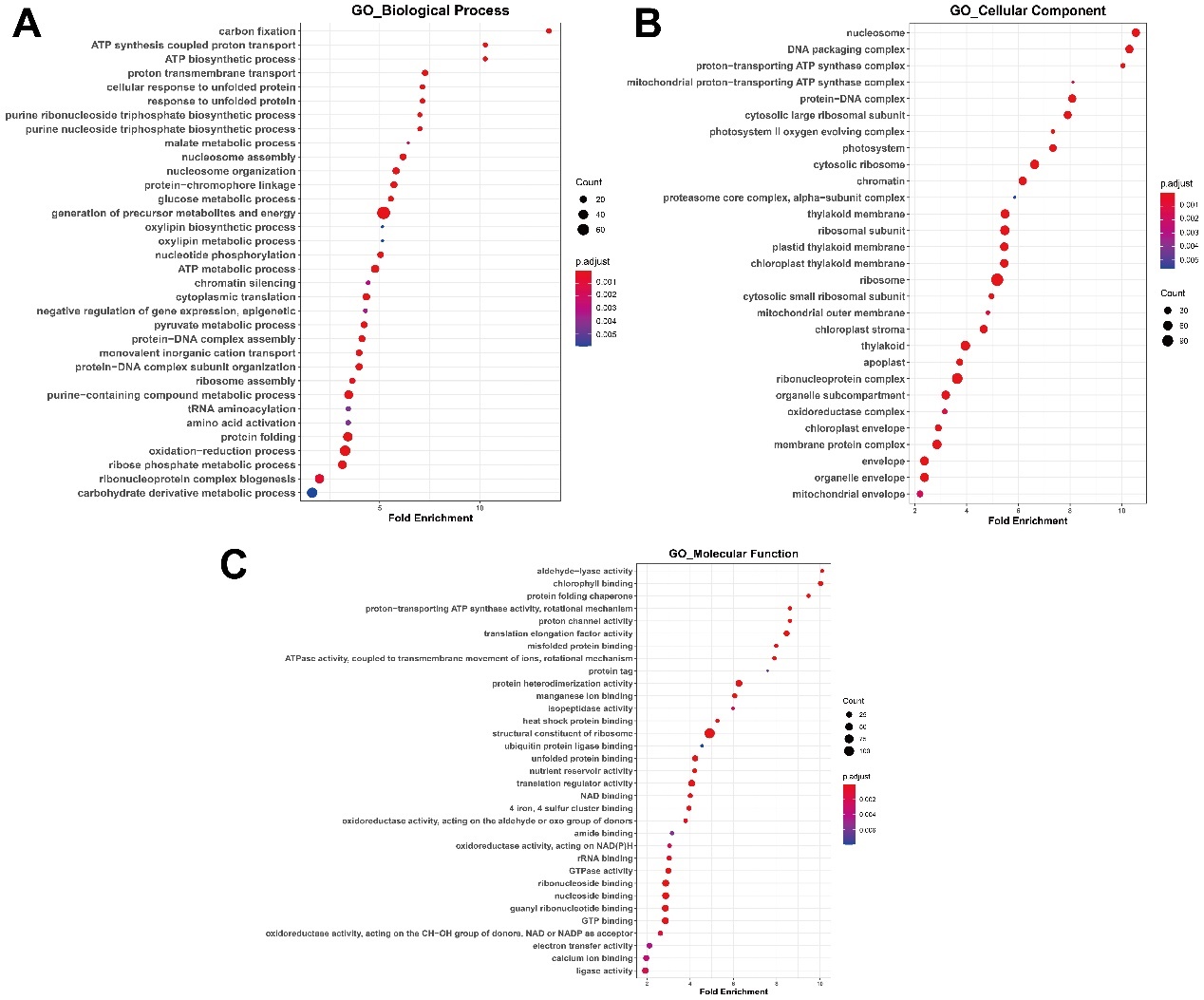
Fig. S3** GO (gene ontology) enrichment analysis of all identified Kac proteins.

**
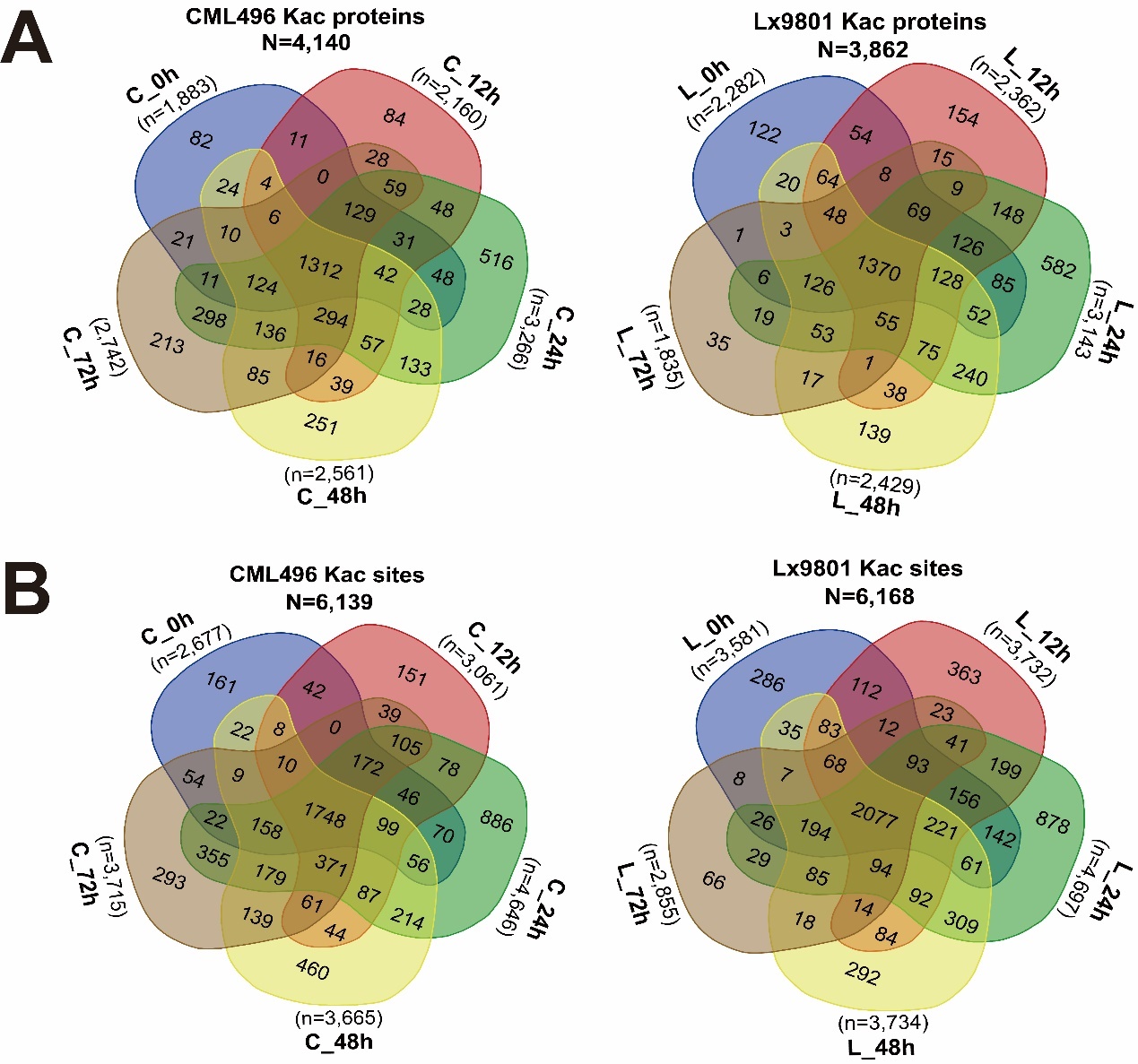
Fig. S4** Distribution of Kac proteins (**A**) and sites (**B**) in SCR-resistant and susceptible maize infected with *P. polysora* for 0-h, 12-h, 24-h, 48-h and 72-h.

**

Fig. S5** Molecular function enrichment analysis of all identified common and specific Kac proteins in CML496 and Lx9801.

**
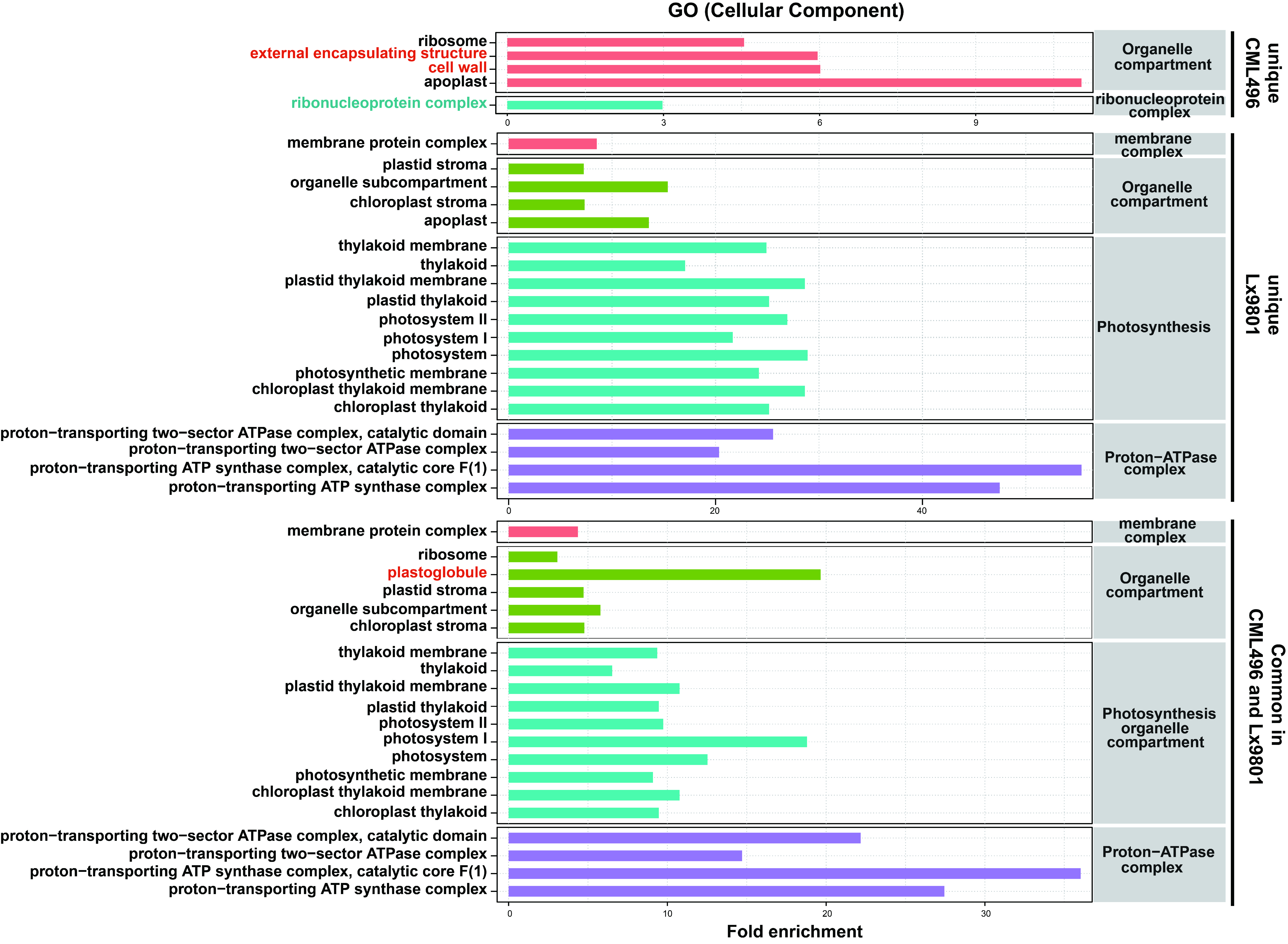
Fig. S6** Cellular component enrichment analysis of all identified common and specific Kac proteins in CML496 and Lx9801.

**
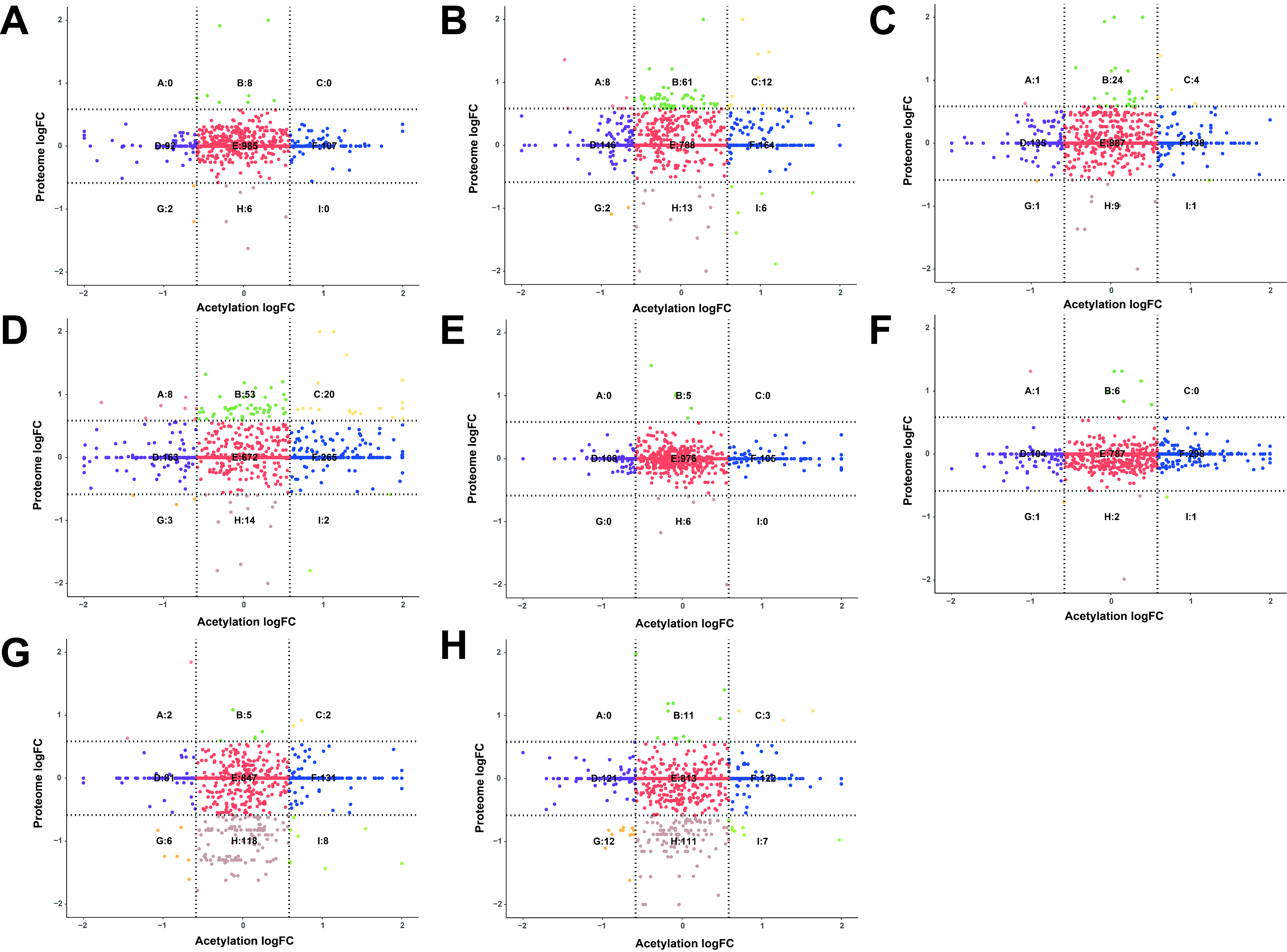
Fig. S7** Fold changes in proteins and Kac sites of CML496 treated with *P. polysora* for 12 h (**A**), 24 h (**B**), 48 h (**C**) and 72 h (**D**) compared to 0 h. Fold changes in proteins and Kac sites of Lx9801 treated with *P. polysora* for 12 h (**E**), 24 h (**F**), 48 h (**G**) and 72 h (**H**) compared to 0 h.

**
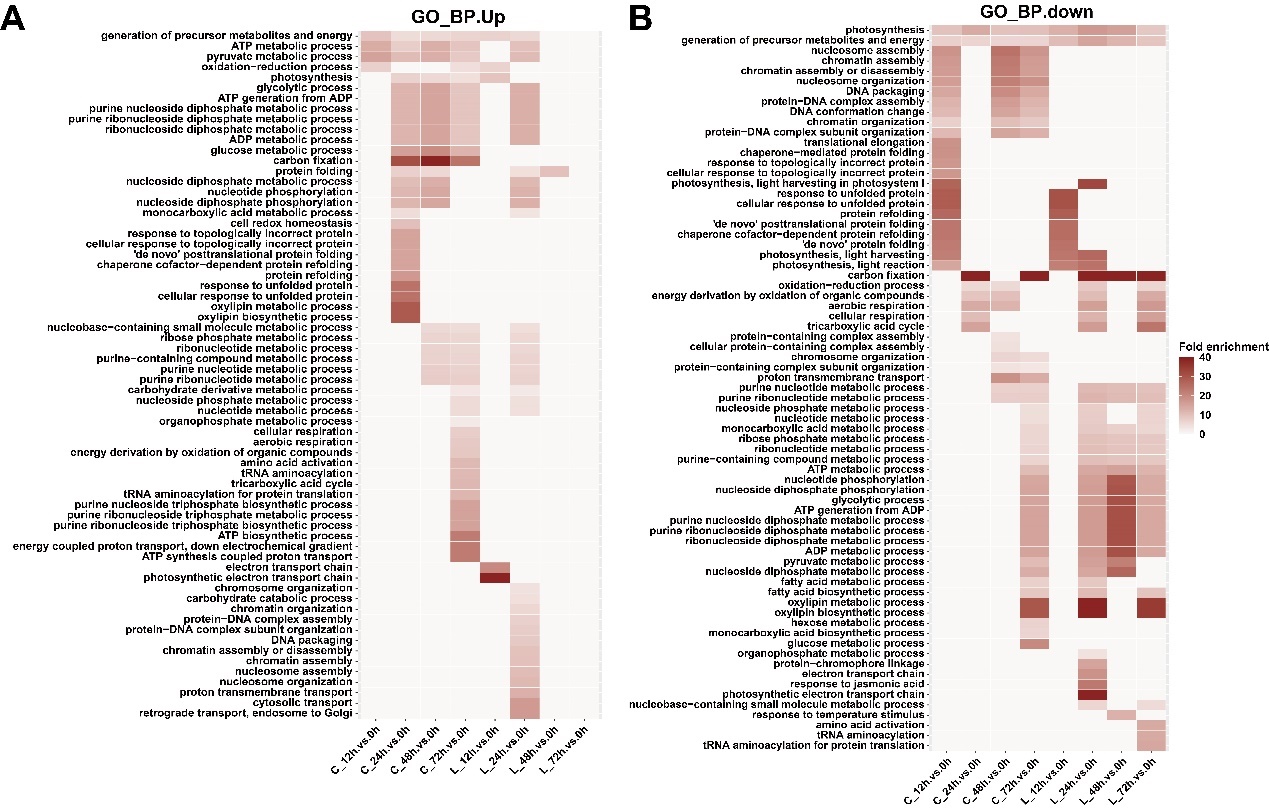
Fig. S8** Biological process enrichment analyses of up-regulated (**A**) and down-regulated (**B**) DKPs in CML496 and Lx9801 with *P. polysora* infection for 12 h, 24 h, 48 h and 72 h compared to 0 h.

**
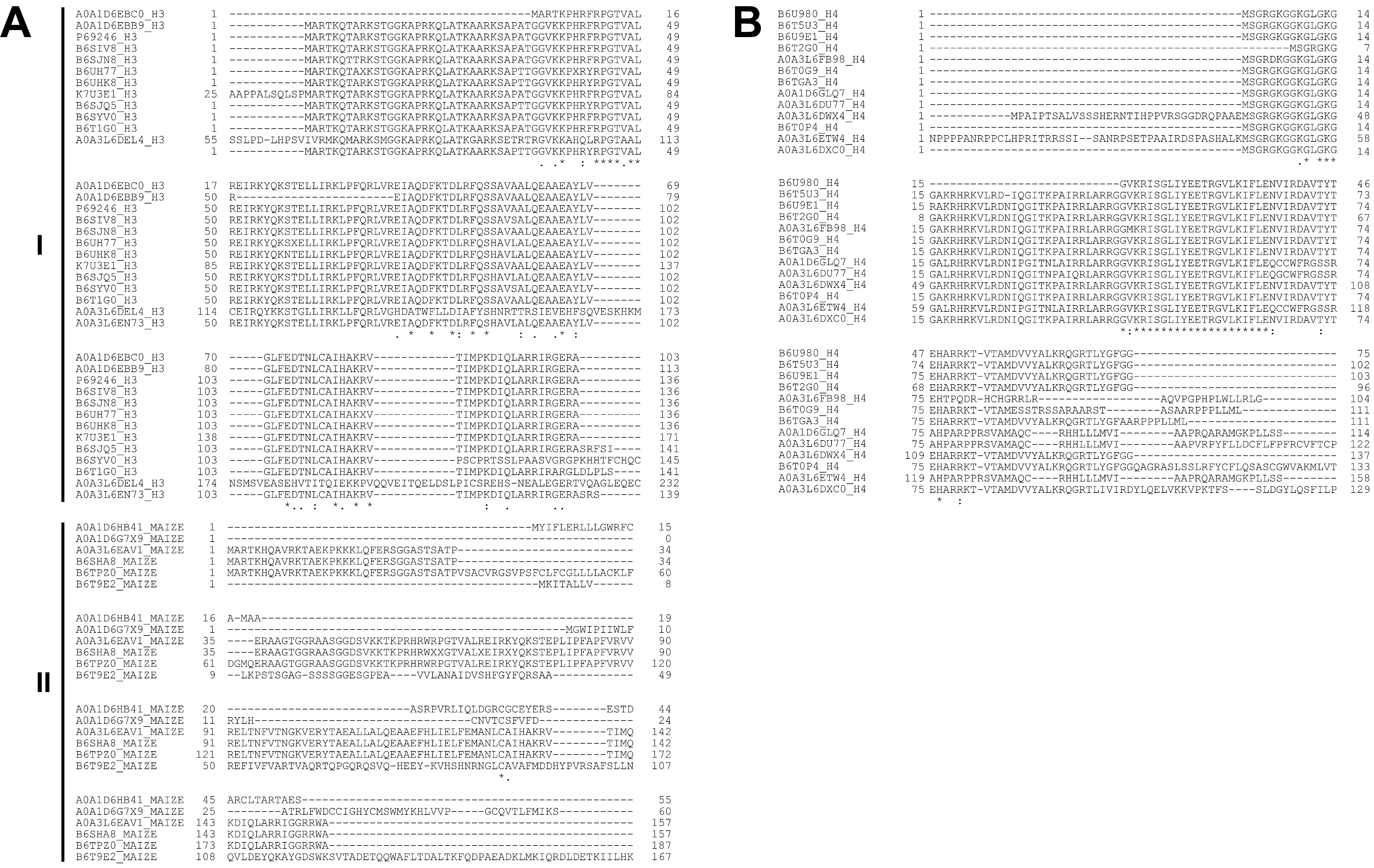
Fig. S9** Sequence alignment of histone H3 (**A**) and H4 (**B**). Asterisks (*) indicate conserved amino acid sites, dots (·) indicate relatively conserved amino acids, colons (:) indicate slightly conserved amino acids.

**
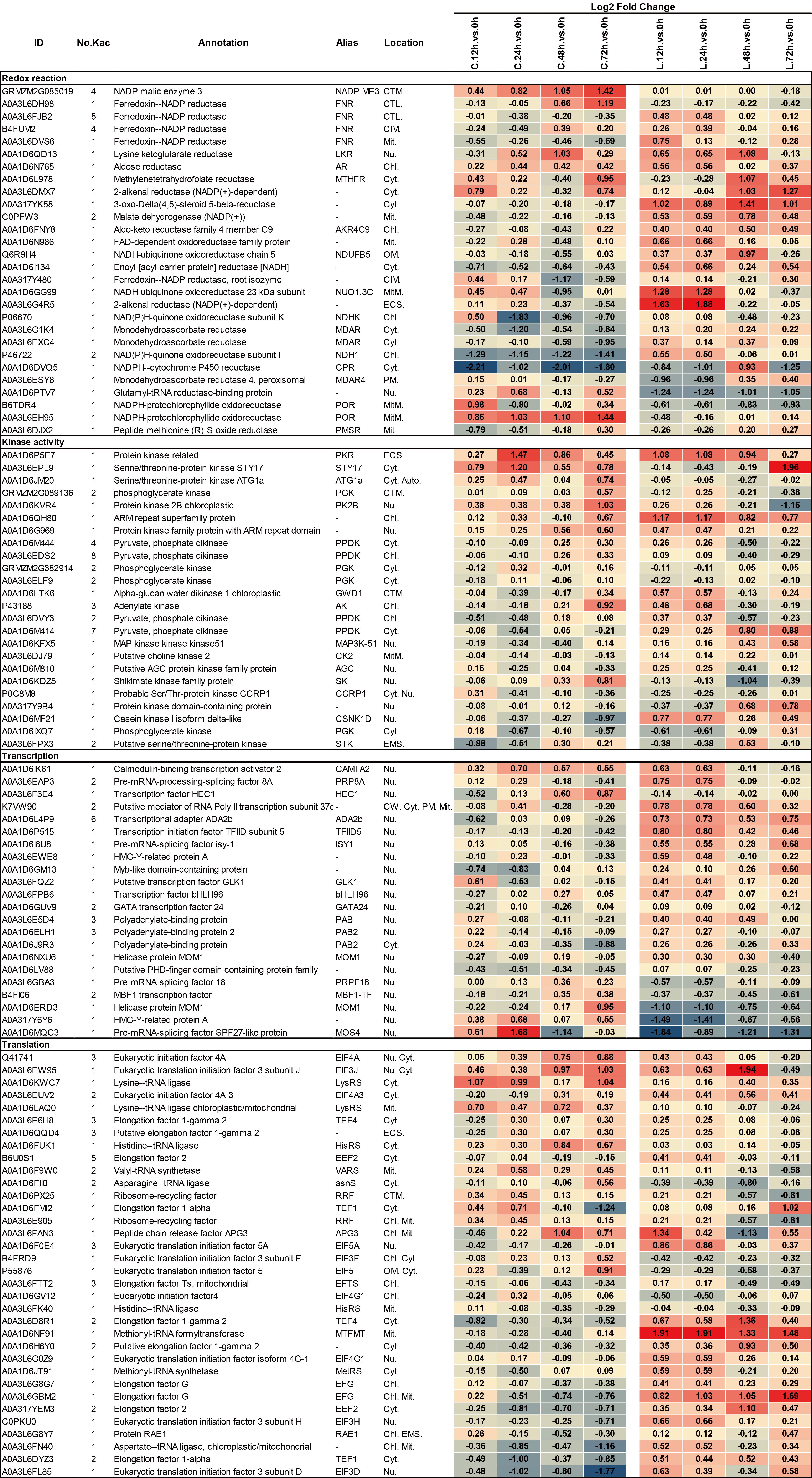
Fig. S10** Heat map of mainly differential acetylated proteins (DAPs) involved in redox reaction, kinase activity, transcription and translation found in SCR-resistant and susceptible maize infected with *P. polysora.*
