# Supplementary figures and images for: A comprehensive dynamic immune acetylproteomics profiling induced by Puccinia polysora in maize
Source: BMC Plant Biol. 2022 Dec 24;22:610. doi: 10.1186/s12870-022-03964-4 (PMC9789614; doi:10.1186/s12870-022-03964-4)

Anti-H3


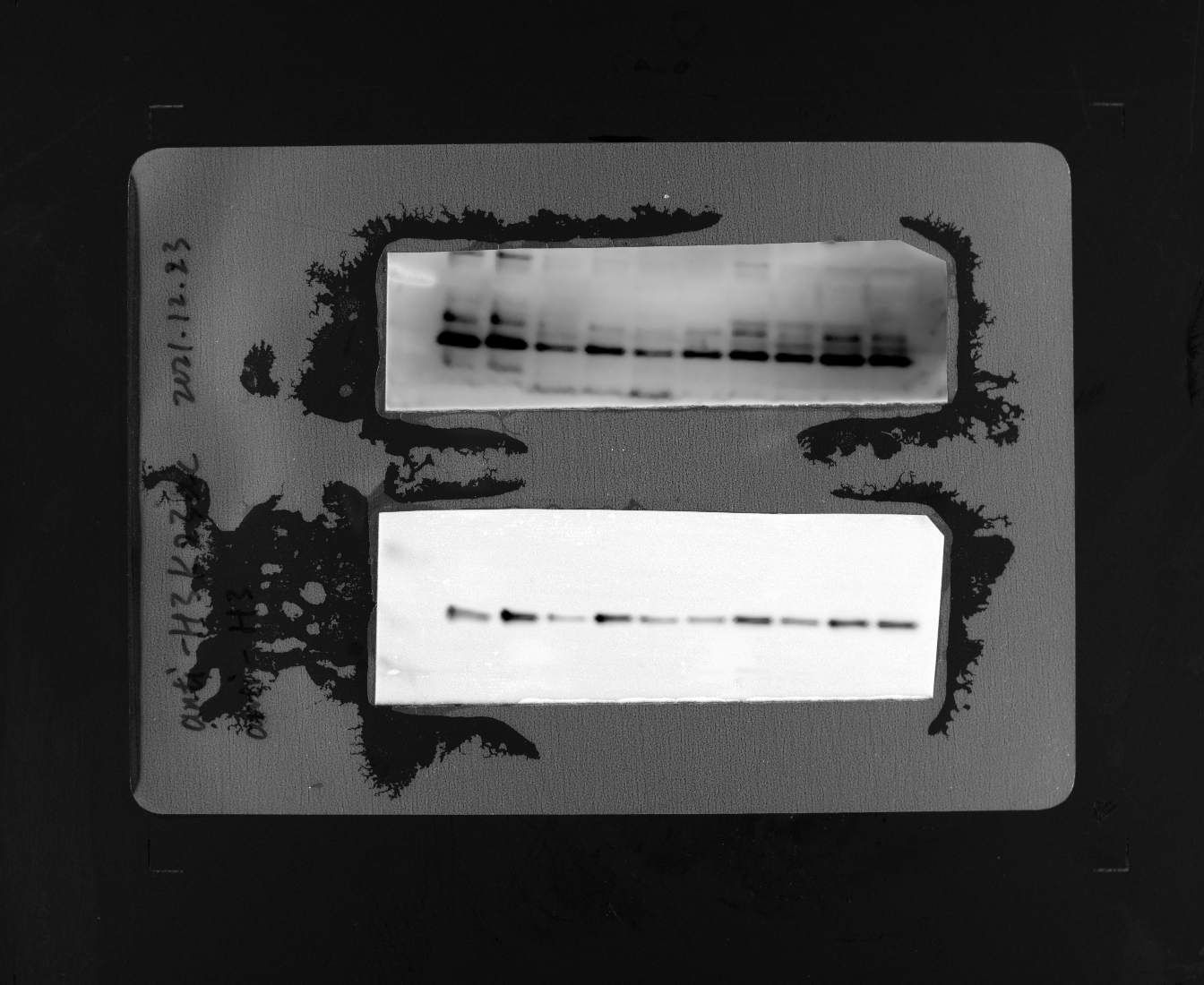


Anti-H3K23ac


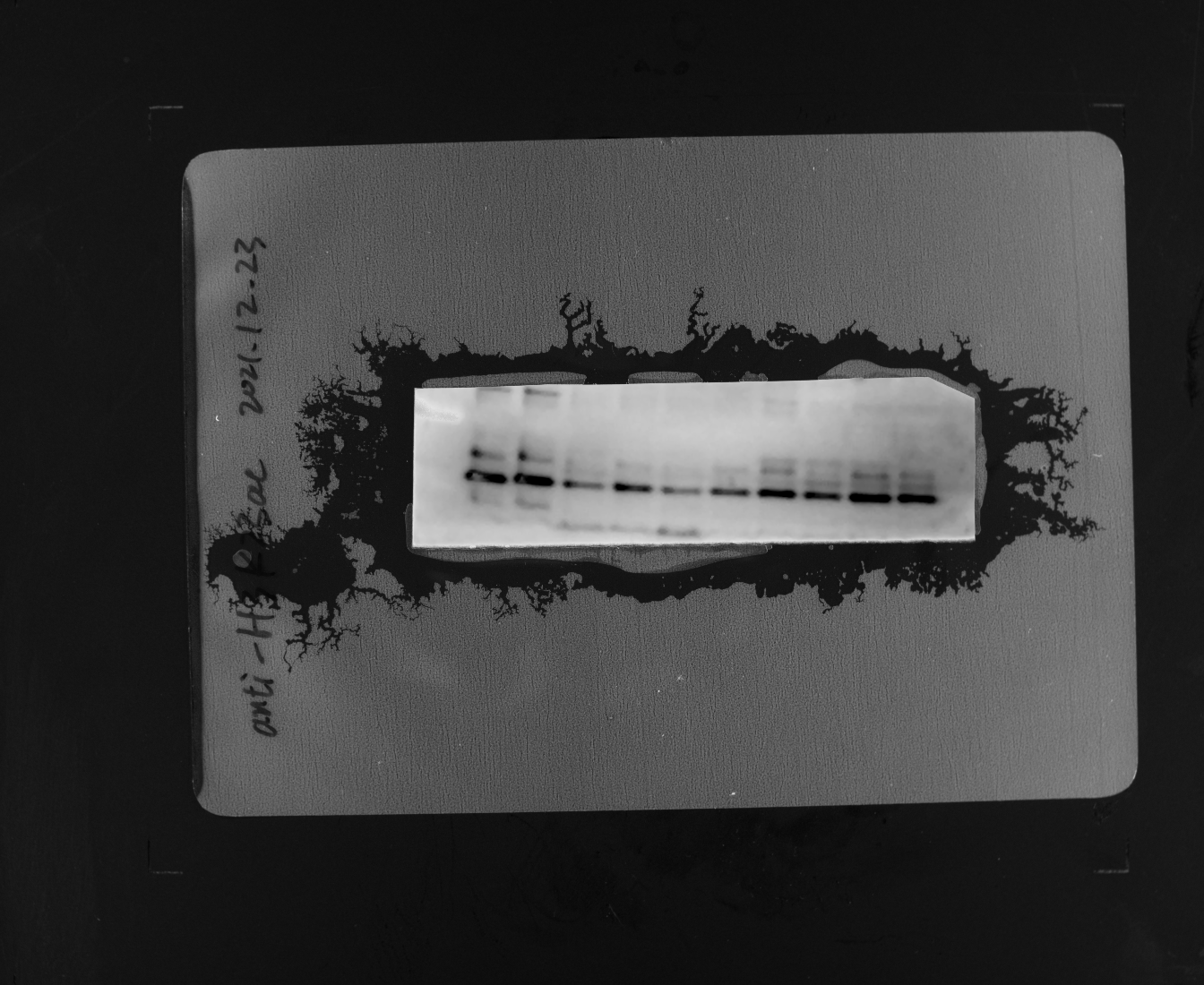


Anti-H3K18ac


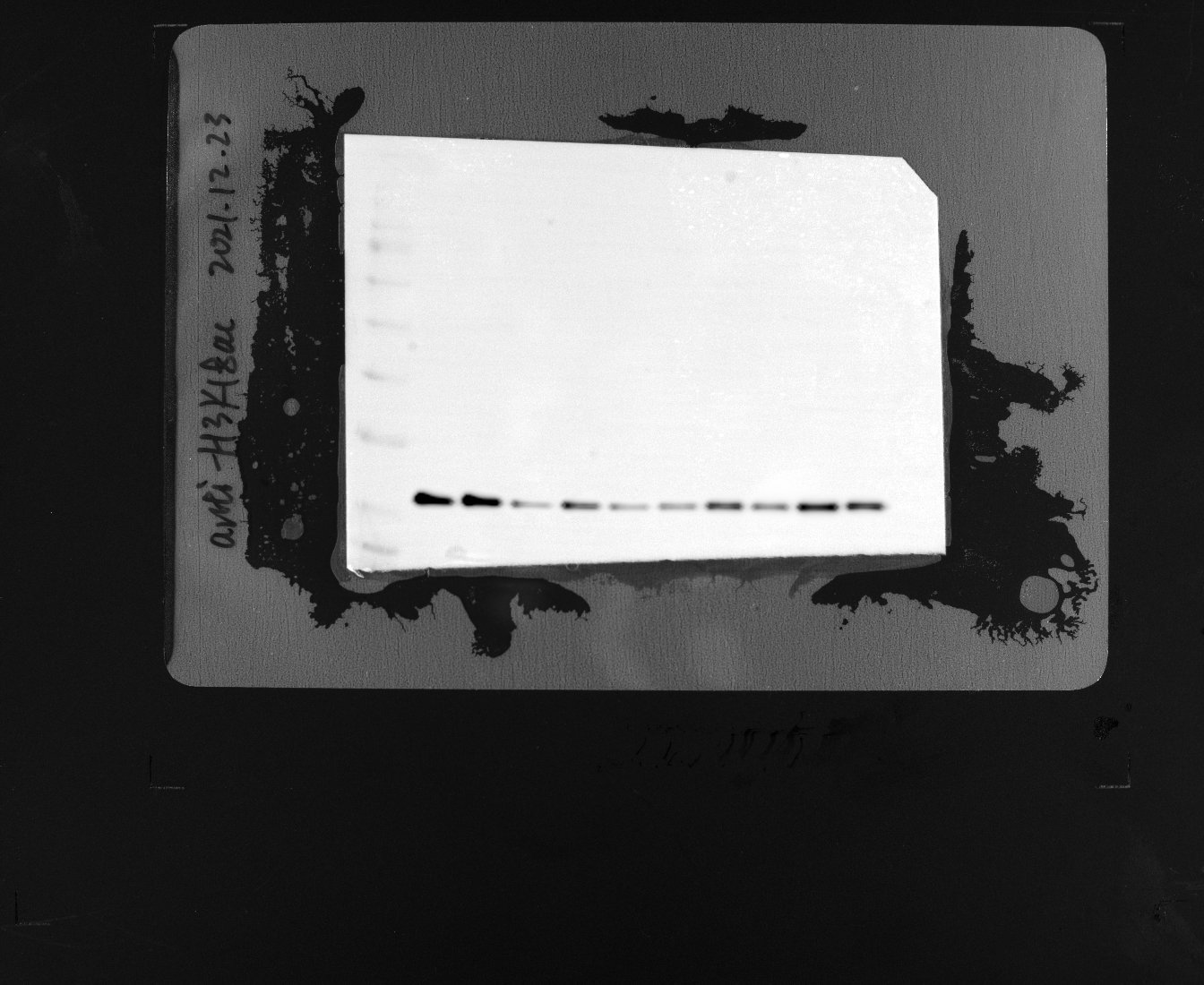


Anti-H3KAC


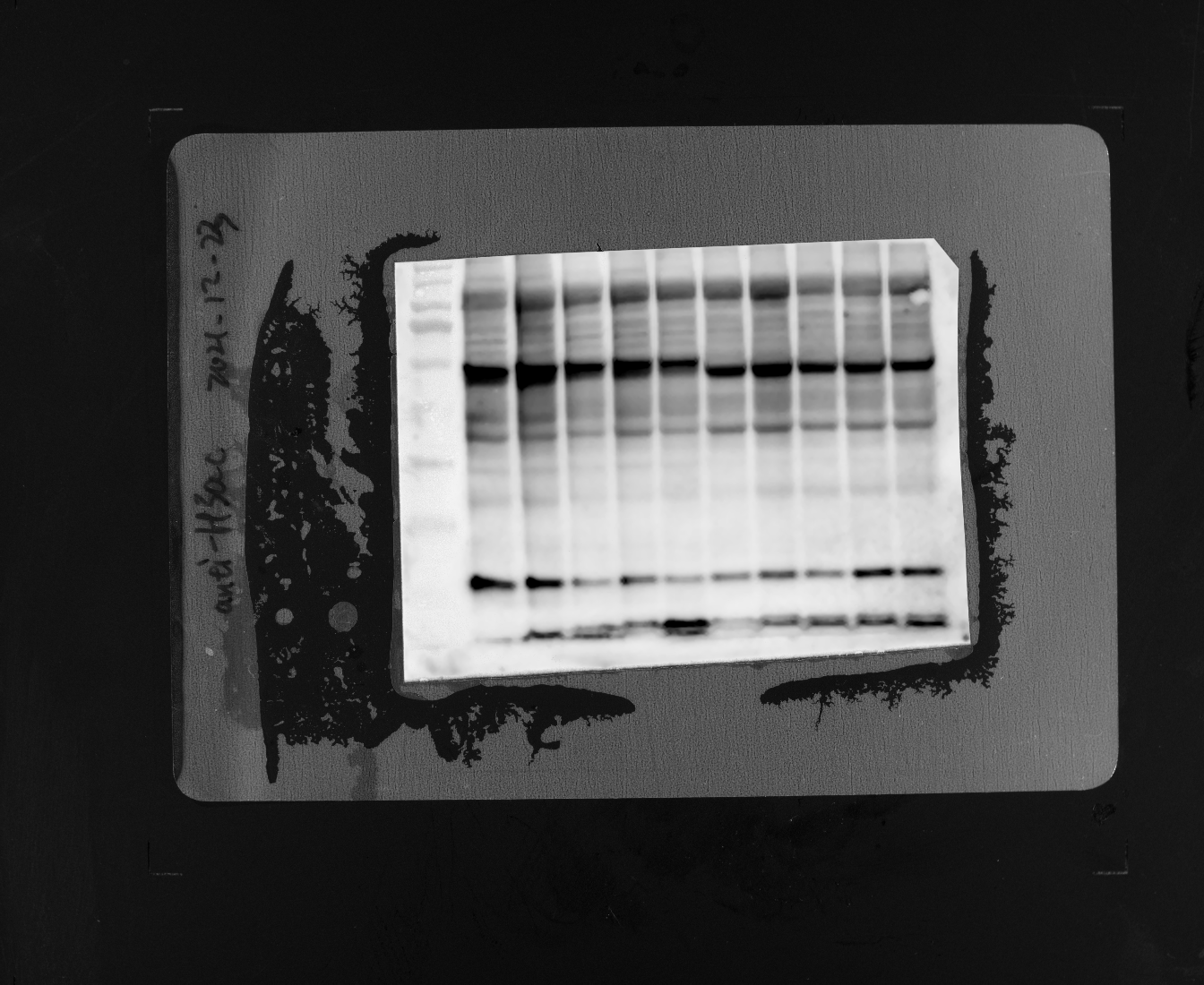


Anti-H4K5ac


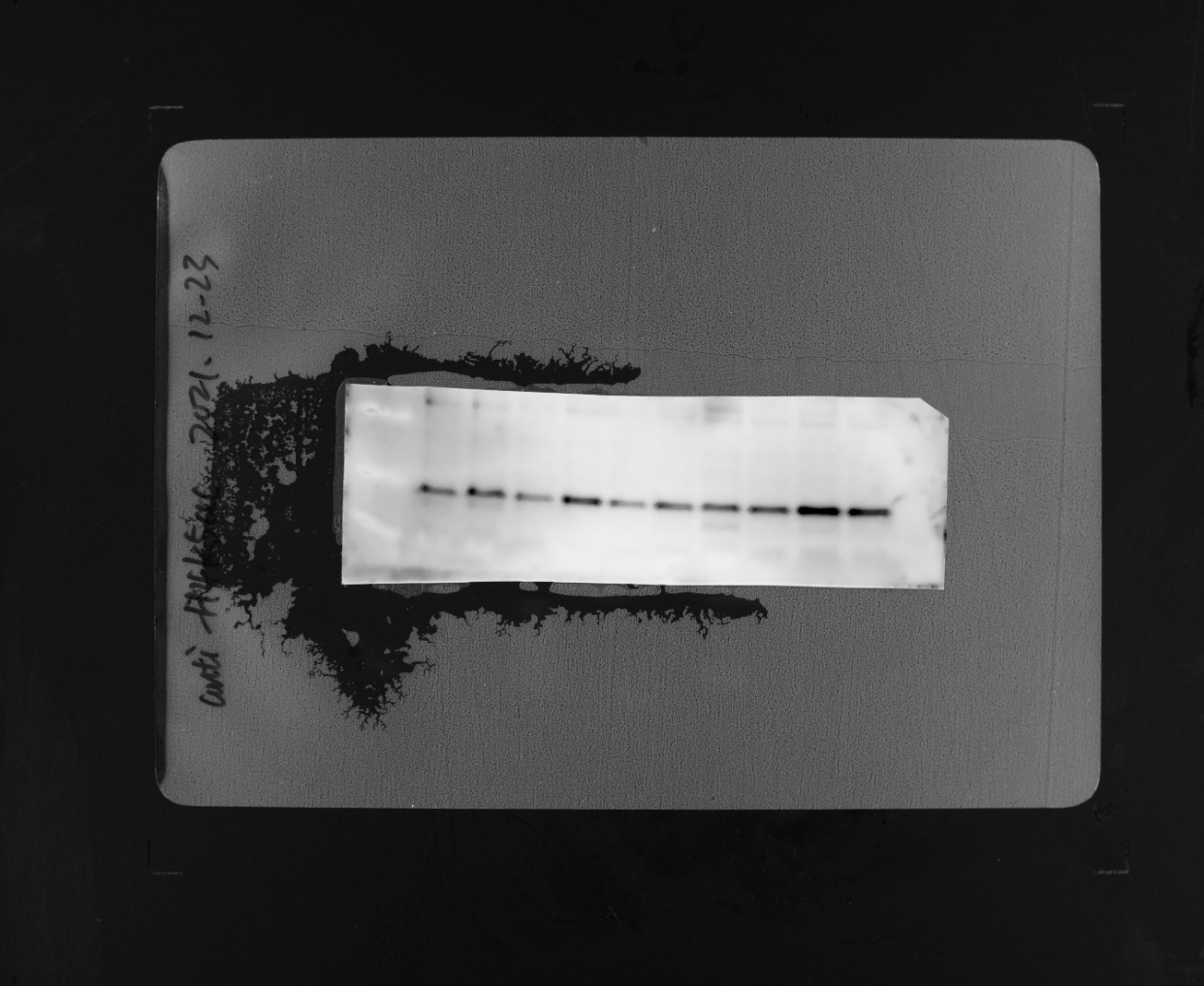


Anti-H4K8ac


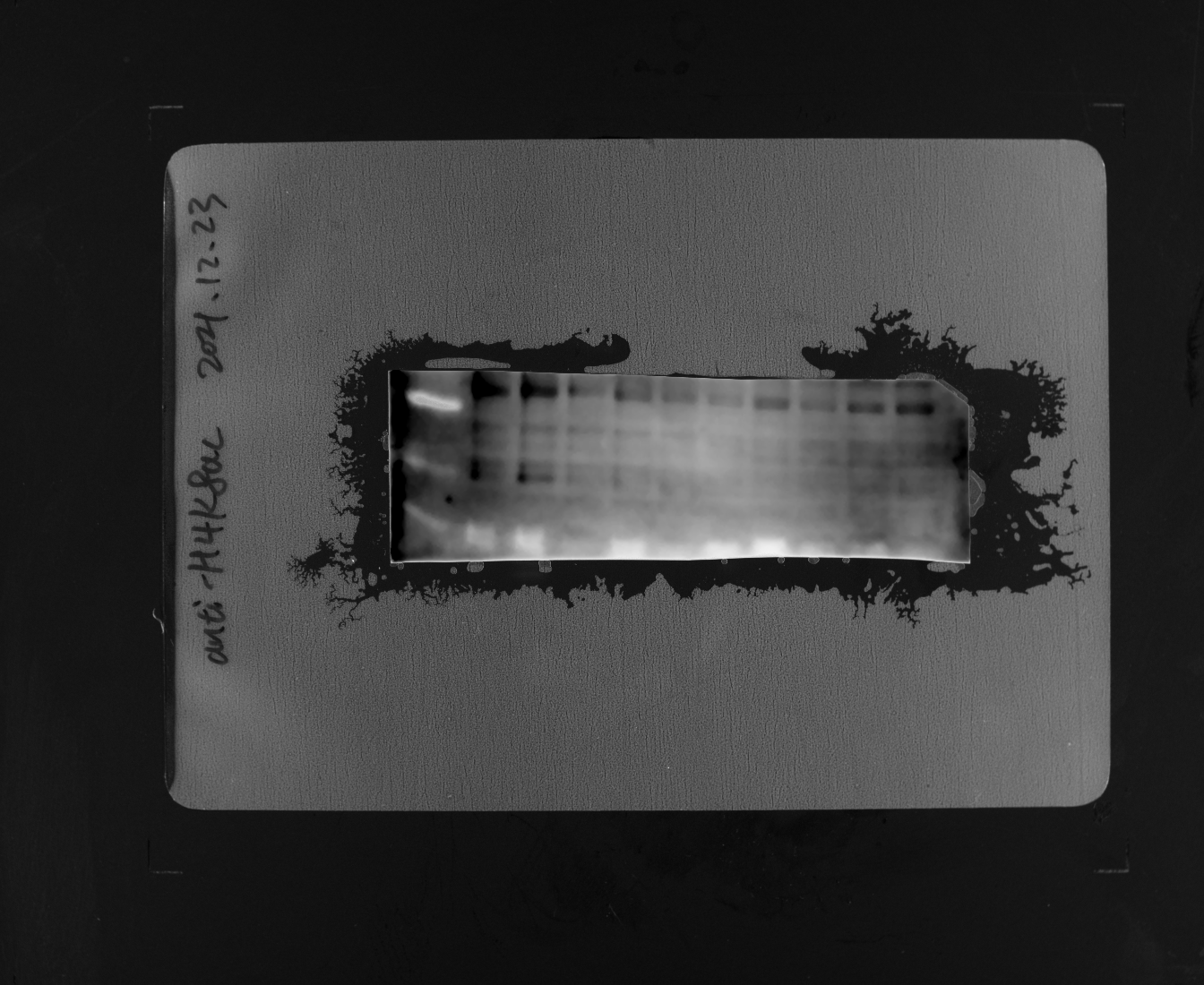


Anti-H4K12ac


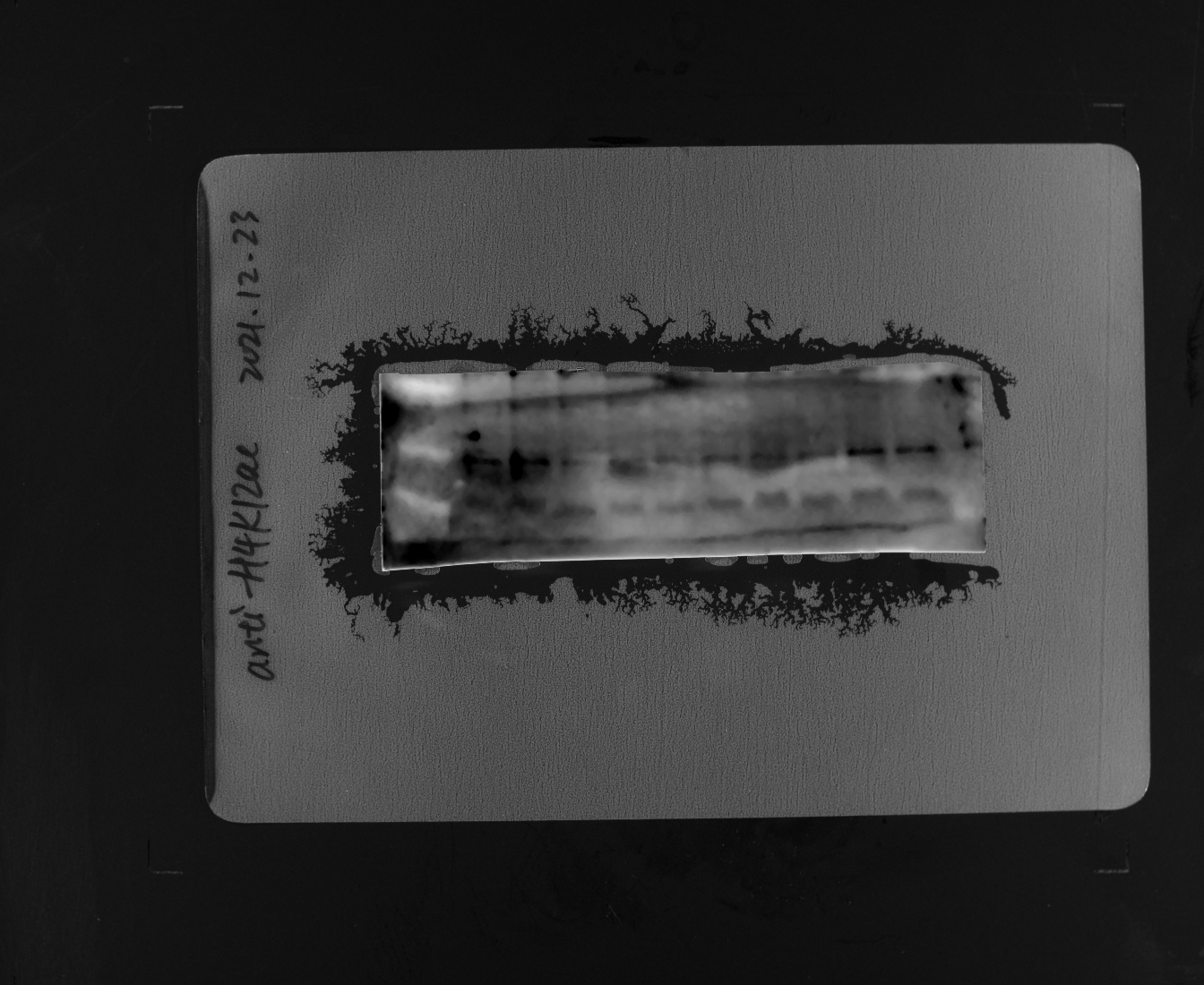


Anti-H4K16ac


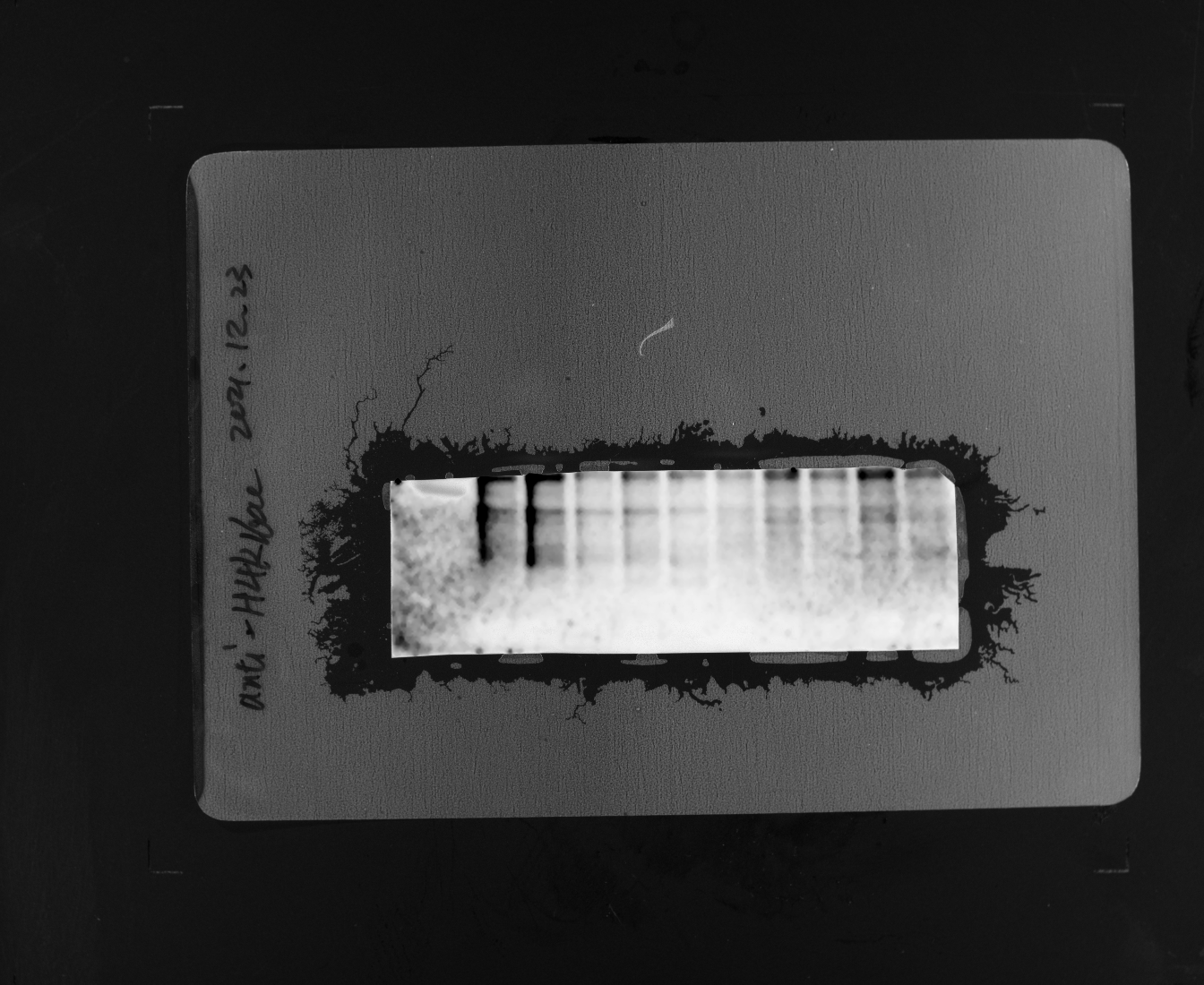

Supplement: Supplementary file 6 — Additional file 6. [file 12870_2022_3964_MOESM6_ESM.docx]
